# Linking leaf economic and hydraulic traits with early-age growth performance and survival of *Eucalyptus pauciflora*

**DOI:** 10.3389/fpls.2022.973087

**Published:** 2022-11-08

**Authors:** João Costa e Silva, Brad M. Potts, Georg Wiehl, Suzanne M. Prober

**Affiliations:** ^1^ Centro de Estudos Florestais, Instituto Superior de Agronomia, Universidade de Lisboa, Lisboa, Portugal; ^2^ School of Natural Sciences, University of Tasmania, Hobart, TAS, Australia; ^3^ Australian Research Council (ARC) Training Centre for Forest Value, University of Tasmania, Hobart, TAS, Australia; ^4^ CSIRO Land and Water, Private Bag 5, Wembley, WA, Australia

**Keywords:** Directional selection, selection gradients, leaf thickness and leaf density, leaf area, stomatal density and stomatal length, vein density, growth performance, tree survival

## Abstract

Selection on plant functional traits may occur through their direct effects on fitness (or a fitness component), or may be mediated by attributes of plant performance which have a direct impact on fitness. Understanding this link is particularly challenging for long-lived organisms, such as forest trees, where lifetime fitness assessments are rarely achievable, and performance features and fitness components are usually quantified from early-life history stages. Accordingly, we studied a cohort of trees from multiple populations of *Eucalyptus pauciflora* grown in a common-garden field trial established at the hot and dry end of the species distribution on the island of Tasmania, Australia. We related the within-population variation in leaf economic (leaf thickness, leaf area and leaf density) and hydraulic (stomatal density, stomatal length and vein density) traits, measured from two-year-old plants, to two-year growth performance (height and stem diameter) and to a fitness component (seven-year survival). When performance-trait relationships were modelled for all traits simultaneously, statistical support for direct effects on growth performance was only observed for leaf thickness and leaf density. Performance-based estimators of directional selection indicated that individuals with reduced leaf thickness and increased leaf density were favoured. Survival-performance relationships were consistent with size-dependent mortality, with fitness-based selection gradients estimated for performance measures providing evidence for directional selection favouring individuals with faster growth. There was no statistical support for an effect associated with the fitness-based quadratic selection gradient estimated for growth performance. Conditional on a performance measure, fitness-based directional selection gradients estimated for the leaf traits did not provide statistical support for direct effects of the focal traits on tree survival. This suggested that, under the environmental conditions of the trial site and time period covered in the current study, early-stage selection on the studied leaf traits may be mediated by their effects on growth performance, which in turn has a positive direct influence on later-age survival. We discuss the potential mechanistic basis of the direct effects of the focal leaf traits on tree growth, and the relevance of a putative causal pathway of trait effects on fitness through mediation by growth performance in the studied hot and dry environment.

## Introduction

Plant functional traits are morpho-physio-phenological traits that impact attributes of plant performance and ultimately fitness (Violle et al., 2007; Caruso et al., 2020b). However, while a plethora of plant functional traits are recognised (Cornelissen et al., 2003; Pérez-Harguindeguy et al., 2013), their associations with plant performance and fitness are variable and may change with scale (i.e. among species, populations within species or individuals within populations), study system, life history stage and environment (Geber and Griffen, 2003; Gómez, 2004; Kingsolver et al., 2012; Siepielski et al., 2017; Warwell and Shaw, 2018; Agrawal, 2020; Caruso et al., 2020a; Laughlin et al., 2020). At the intra-specific level, functional traits are the basis of local and future adaptation (Hendry, 2017; Baughman et al., 2019; Ahrens et al., 2020) and, for a given species or population, it is important to understand the specific traits directly affecting plant performance and ultimately fitness (Caruso et al., 2020a). Indeed, with climate change trends in the Anthropocene, there is a focus on traits that confer fitness advantages in hotter and drier conditions (Groom et al., 2004; Choat et al., 2018). This is particularly relevant to forest tree species, where reports of drought/heat induced forest decline are increasing (Brodribb et al., 2020; Lloret and Batllori, 2021; Hammond et al., 2022). However, establishing fitness-trait relationships is particularly difficult for long-lived organisms such as forest trees (Petit and Hampe, 2006; Alberto et al., 2013; Laughlin et al., 2020) where the evaluation of lifetime fitness (e.g. total number of zygotes produced - Walsh and Lynch, 2018) is impractical, but usefully addressed through the study of its components (Orr, 2009; Franklin and Morrissey, 2017; Walsh and Lynch, 2018). Accordingly, quantitative inferences about natural selection acting on traits of adaptive value have been based on fitness components (e.g. tree survival and/or fecundity), usually assessed during early stages of the life cycle (Gómez, 2004; Ramírez-Valiente et al., 2014; Costa e Silva et al., 2018; Warwell and Shaw, 2018; Warwell and Shaw, 2019; Costa e Silva et al., 2021; Ramírez-Valiente et al., 2021).

Formal quantitative inference of natural selection is usually made by studying the relationship between fitness (or a fitness component) and a multivariate phenotype (defined by the focal traits subject to selection) assessed on the same individuals, providing *fitness-based* selection gradients (Lande, 1979; Lande and Arnold, 1983; Walsh and Lynch, 2018). However, for a given study system, it may be challenging to assess fitness and traits on the same individuals in the wild. In this context, using path analytical methods, Arnold (1983) introduced the notion of *performance-mediated* selection gradients, where selection acting on the focal traits is mediated by their effects on attributes of organismal performance, which in turn are expected to directly influence fitness or a fitness component (see also Arnold, 2003). For a pathway connecting traits to fitness via a performance variable, selection gradients are thus obtained by the product of regression coefficients estimated from the modelling of performance-trait and fitness-performance relationships, with the advantage that the data used to build these regression models could come from different cohorts of individuals as, for example, separate studies within a species (Arnold, 1983). When field assessments of fitness cannot be achieved, an alternative is to model a fitness proxy (such as a growth measure) that is expected to have a (positive) causal effect on fitness or a fitness component (Franklin and Morrissey, 2017). This third approach leads to a *performance-based* estimator of selection, which effectively refers only to the part entailing the performance-trait relationship in the Arnold (1983) framework.

As highlighted by Franklin and Morrissey (2017), performance-based estimators of selection may not be reflective of fitness-based or performance-mediated selection gradients, as fitness proxies cannot be assumed to represent the demographic contribution of individuals to future generations. However, studies that accomplish the evaluation of both the influence of the focal traits on individual performance and the effect of individual performance on fitness will be important to gain a broad view of selection and adaptation (Arnold, 1983). In addition, when data are available on the same individuals for the focal traits, performance variables and fitness components, it may be possible to use the information from the modelling of performance-trait, fitness-performance and fitness-trait relationships to examine the conditions under which it is plausible to utilize performance as a substitute for fitness in selection analysis, as demonstrated by Franklin and Morrissey (2017). The present study evaluated these relationships in *Eucalyptus pauciflora*, using within-population variation in leaf economic and hydraulic functional traits, two growth performance attributes (height and diameter) and a fitness component (tree survival), measured in a common-garden (multi-population) field trial established at the hot and dry end of the species distribution on the island of Tasmania, Australia (Costa e Silva et al., 2019; Prober et al., 2022).

*Eucalyptus pauciflora* is one of the main tree species used for ecological restoration in the dry Midlands region of Tasmania (Bailey et al., 2021). Climate change models predicted that increasing temperatures and evapotranspiration will result in most Tasmanian *E. pauciflora* populations being at risk of maladaptation by 2080 (Harrison, 2021). Relative to a contemporary climate base (1976-2005), these climate changes are already detectable (Harrison, 2021) and, in terms of average maximum temperature, the growing period climate at the common-garden trial examined in the current study is already outside of the upper contemporary range of all field-tested populations (see Figure 6 in Costa e Silva et al., 2019). While acute heat or drought events have yet to impact the trial, we hypothesised that putative directional selection acting on functional traits would favour individuals with phenotypes more adapted for growth under hotter, seasonally dry climates. In the current study, we focused on leaf economic and hydraulic traits, which underlie the structural investment required for photosynthesis and characteristics affecting leaf gas exchange and water transport, respectively, some of which show significant population variance in *E. pauciflora* (Prober et al., 2022). Such traits have been extensively studied in plants (Sack et al., 2013; Reich, 2014), and often have shown an association with variation in home-site habitat and plant performance at both the between species (Niinemets, 2001; Reich, 2014; Blackman et al., 2018) and, increasingly, within species (Warren et al., 2006; McLean et al., 2014; Anderegg et al., 2018; Ahrens et al., 2020) levels. For individual phenotypic variation within populations of *E. pauciflora*, we here explore the following questions:

- Which leaf traits have a direct effect on growth performance at an early age?
- Is early-age growth performance related to later-age tree survival or, in other words, is there size-dependent mortality? If so, is the studied fitness component affected differently by the measured growth performance attributes?
- Do the leaf traits have direct fitness consequences, or is the influence of the traits on the fitness component mediated through their effects on growth performance?

## Materials and methods

### Plant material

Detailed descriptions of the *E. pauciflora* plant material and the common-garden field trial studied are given in Costa e Silva et al. (2019), Bailey et al. (2021) and Prober et al. (2022). The plants analysed were growing in a common-garden trial that was established on an ex-pasture site adjacent to remnant native forest at Connorville in northern Tasmania, Australia (41.828˚S, 147.138˚E, altitude of 185 m). The soil was ripped and mounded, with rip lines 3 m apart (Davidson et al., 2021a). The planted seedlings were from 318 families derived from single-tree, open-pollinated seed collections. The mother trees were located in 38 native populations, sampled from throughout the Tasmanian distribution of *E. pauciflora* (Gauli et al., 2014). Within the trial, families were arranged in single-tree plots in each of 8 blocks. Family arrangement within a block followed a resolvable row-column experimental design, and thus the sampled populations can be considered as randomly positioned within each block. Our study was based on a subsample of one plant from each of 36 populations and each of 5 blocks. A unique family was sampled in each block, resulting in a population being represented by one plant from each of five, well-separated mother trees in their native population. Therefore, the cohort of individuals sampled for the current study consisted of 180 plants (36 populations x 5 plants per population).

### Measurements

Sampling of leaves for measuring the functional (economic and hydraulic) leaf traits, and the assessment of growth performance attributes, were undertaken at age 2 years from planting (June 2016) when the trees were in the non-reproductive stage. **Supplementary Table 1** provides the estimated means and standard deviations for these measures. Tree survival was assessed at age 7 years from planting, and it was used as a fitness component.

#### Leaf economic and hydraulic traits

Details on the sampling and measurement of leaves are provided in Prober et al. (2022). In brief, thirteen mature, fully-expanded leaves (excluding the petiole) were collected from three branches distributed around the mid-outer, sun-exposed part of the canopy of each tree. Leaf economic traits were measured from ten leaves, and the more time-consuming leaf hydraulic traits were measured from the remaining three leaves. The leaf economic traits measured were leaf thickness (mm), leaf area (cm^2^) and leaf density (mg/mm^3^). Leaf thickness was measured using digital callipers, avoiding the influence of major leaf veins; leaf area was measured from scanned images obtained by using a digital scanner; and leaf density was calculated from leaf area, leaf thickness and dry weight. The leaf hydraulic traits measured were stomatal density (number/mm^2^), stomatal length (μm) and vein density (mm/mm^2^). Stomata observations were made with a microscope by using the nail polish impression method, as described in Franks et al. (2009). Stomatal density (i.e. number of stomata per unit epidermal area) was calculated for each leaf as the mean of six fields of view (three on each side of each leaf). Stomatal length for each leaf was the mean of the guard cell length measured from 20 stomata complexes (10 guard cell pairs on each side of each leaf). Vein density measurements followed the general protocols described in Brodribb et al. (2013). The leaf-level data were averaged to give a single value of each trait for each tree.

#### Growth performance

Growth performance was assessed using maximum tree height and stem diameter, commonly used measures of the productivity of forest trees (West, 2009). Tree height (m) was measured with height poles, and the stem diameter (mm) was measured using a diameter tape at 50 cm above-ground level, as detailed in Davidson et al. (2021b).

#### Tree survival

The fitness component studied at an early life-history stage was the time-averaged survival between 2 and 7 years from planting. At seven years from planting, trees of the sampled cohort were scored as a binary outcome whereby a tree was classified as dead (scored as 0) when no live plant tissue was evident above ground or the plant was in very poor vegetative health, otherwise it was classified as alive (scored as 1). Of the sampled trees, 70% survived to the final assessment. Yet, while pertaining to the 5-year interval, observations showed that virtually all the recorded mortality occurred between ages 4 and 7 years.

### Data analysis

Our experimental observations have a two-level hierarchical structure, where individual trees are clustered into seed-source populations. We favoured a fixed-effects (FE) model specification in the data analysis, as our interest was focused on the estimation and statistical inference for within-population effects of continuous predictors (hereafter also referred to as “covariates”). This approach accommodates the dependence among observations that may occur when they are clustered into higher-level groups, while also preventing any bias on regression coefficients of lower-level covariates due to omitted variables at the higher level (Allison, 2009; Bell et al., 2019) (see **Supplementary Methods 1** for the application of fixed-effects versus random-effects linear models to our data). The SAS 9.4 software (SAS, 2017) was used for data analysis.

#### Relationship of growth performance with functional traits

Performance-trait relationships were studied by estimating and testing the effects of focal functional traits on a growth performance measure, under the following general linear model:


(1) $$y_{ijk}\;=\;\beta_{0}\;+\;u_{j}\;+\;r_{k}\;+\;\sum_{h=1}^{p}\beta_{h}x_{h_{ijk}}\;+\;\;\varepsilon_{ijk}$$

where *y_ijk_* is the observation on a measure of growth performance (height or diameter) for the *i^th^* individual tree within the *j^th^* population (*j* = 1,…, 36) at the *k^th^* block (*k* = 1,…, 5); *β_0_* is an intercept term; *u_j_* is the fixed effect of the *j^th^* population; *r_k_* is the fixed effect of the *k^th^* block; *x*_*h*__*ijk*___ is the value of the *h^th^* covariate (*h* = 1,…, *p*), pertaining to the observation on a functional trait for the *ijk^th^* individual; *β_h_* is the regression parameter for the *h^th^* covariate, representing the average within-population effect of a functional trait on the performance measure; and *ε_ijk_* is a random residual.

Scaling in relation to the grand mean is both permissible and meaningful for the studied functional traits (Houle et al., 2011). Thus, the *x*_*h*__*ijk*___ data in Equation (1) were mean standardized, and then expressed on a (dimensionless) percentage scale for comparison of regression parameter estimates (i.e. ${\hat{\beta }}_{h}$ will refer to the estimated effect on performance from increasing the trait’s covariate values by 1%). The performance measure was also relativised via the division by its grand mean to derive a proxy for relative fitness (Franklin and Morrissey, 2017). In this sense, ${\hat{\beta }}_{h}$ can be interpreted as a within-population, performance-based average estimate of directional selection operating on the *h^th^* trait, calculated by combining data across populations sampled from the distributional range of *E. pauciflora* in Tasmania.

For either performance response variable, least-squares estimation of model parameters and their standard errors were undertaken for specifications of Equation (1) that included: each trait individually; the three traits within the economic or hydraulic sets; and all the six functional traits across both sets. Statistical inference about the effect of *h^th^* covariate on a performance measure was provided by a (partial) *t*-test on the estimated *β_h_* coefficient, and by computing the associated 95% confidence interval. Residual plots and other diagnostic statistics indicated that the model assumptions were met (i.e. normality of residual distributions, no residual heteroskedasticity, absence of statistically significant outliers, and variance inflation factors < 2).

#### Relationship of tree survival with growth performance

We approached size-dependent mortality from the perspective of evaluating the strength and form of selection acting on growth performance, via its influence on time-averaged (i.e. over the five-year period studied) tree survival used as a fitness component. Using individual variation within populations, a generalized regression analysis was applied to model the dependence of tree survival on individual performance values, and subsequently derive (fitness-based) selection gradient parameters for performance. Under the assumption of only directional selection acting on performance, we first modelled a linear term for a performance variable in the generalized linear FE model defined as:


(2) $$g(E(y_{ijk}{\text{ | x}}_{ijk}))\;=\;g(\mu_{ijk})\;=\;\beta_{0}\;+\;u_{j}\;+\;r_{k}\;+\;\beta_{1}x_{ijk}\;$$

where *y_ijk_* is the observation on an individual’s survival (i.e. a binary outcome of 0 or 1), *g*(.) is a link function relating the conditional expectation [*E*(*y*_*ijk*_ | **x**_*ijk*_) = *μ*_*ijk*_] of the binary response to the linear predictor *η*_*ijk*_ = *β*_0_  +  *u*_*j*_  +  *r*_*k*_  +  *β*_1_ *x*_*ijk*_ ; **x**_*ijk*_ is a row vector for an individual from the design matrix **X** of all the explanatory variables included in the linear predictor; *β_0_*, *u_j_* and *r_k_* are defined as before; *x_ijk_* is the value of the performance variable - height or diameter - for the *ijk^th^* individual; and *β*_1_ is a regression parameter for the effect of performance on survival. The binary responses were assumed to be Bernoulli distributed with a parameter *μ_ijk_*, which is the conditional probability of tree survival [i.e. *P*(*y*_*ijk*_ = 1 | **x**_*ijk*_) ] in our case; *g*(.) used the logit link function, such that $P(y_{ijk}\;={1 |\text{x}}_{ijk})\;=\;g^{-1}(\eta_{ijk})\;=\;\frac{exp{(\text{x}}_{ijk}\beta )}{1+\;exp{(\text{x}}_{ijk}\beta )\;}$ where *g* ^-1^(.) is the inverse of *g*(.), *exp* denotes the exponential function, and ***β*** is a column vector of coefficients comprising all fitted parameters. The *x_ijk_* in Equation (2) refer to observations that were mean standardized through their division by the grand mean $\left(\bar{x}\right)$ of the performance variable (so that the regression parameter estimates will pertain to changes on a comparable, percentage scale for either performance measure). Allowing for the possibility of quadratic selection on growth performance involved the extension of Equation (2) to include a quadratic term $\beta_{2}{\left(\frac{x_{ijk}\text{- }{\bar{x}}_{j}}{\bar{x}}\right)}^{2}$ for the focal performance covariate in the definition of *η_ijk_*. In this context, note that the *x_ijk_* data were population-mean centered (by subtracting the ${\bar{x}}_{j}$ mean from all observations belonging to the *j^th^* population), in order to avoid multicollinearity and to obtain covariate values for the estimation of a within-population, nonlinear effect of growth performance on the logit scale (log-odds of survival).

Reflecting few populations where all trees were either alive or dead, quasi-complete separation was detected in the data analysis, indicating that classical maximum likelihood estimation of model parameters and associated statistical inference would not be reliable. Consequently, the bias-reducing penalized maximum likelihood method developed by Firth (1993) was used for estimation of the model parameters, and 95% profile penalized-likelihood confidence intervals were used for statistical inference about the regression parameters on the logit scale (Heinze and Schemper, 2002; Heinze, 2006). The predictive ability of a specified logistic model was evaluated by the Tjur’s coefficient of discrimination (Tjur, 2009).

Derivative-based estimates of selection gradients (e.g. Janzen and Stern, 1998; Morrissey and Sakrejda, 2013; Franklin and Morrissey, 2017; Costa e Silva et al., 2021) were obtained for either performance measure from a given fitted logistic model. An average directional selection gradient was derived under both definitions of *η_ijk_* (with a linear term, or with both linear and quadratic terms, for performance), whereas an average quadratic selection gradient was derived only when *η_ijk_* had a quadratic term for performance. Besides being summary measures of the strength and form of selection, these derivative-based estimates of directional and quadratic selection gradients describe the average slope and curvature, respectively, of the individual selection surface on the expected fitness scale (Walsh and Lynch, 2018), given the observed range of performance values at age 2 years and the subsequent period during which tree mortality has occurred. Under this geometric interpretation, the quadratic selection gradient estimated by averaging individual second partial derivatives was not multiplied by a factor of 2 (as would be required for a least-squares estimate of a quadratic regression coefficient to obtain an equivalence with a quadratic selection gradient - Stinchcombe et al., 2008). The methods used to calculate partial derivatives and to estimate average selection gradients are detailed in **Supplementary Methods 2**. In particular, analytical expressions (i.e. S2_5 and S2_6) of first and second partial derivatives are provided for the estimation of average directional and quadratic selection gradients when a logistic model includes both linear and quadratic terms for a focal covariate, as the approach described by Janzen and Stern (1998) should not be used in this case. A non-parametric cluster bootstrapping procedure was performed to obtain the standard errors for the selection gradient estimates, and to provide the corresponding bias-corrected and accelerated (BCa) 95% confidence intervals (for further details, see **Supplementary Methods 3**). Statistical support against a null hypothesis being true (i.e. against the absence of an effect associated with a selection gradient) was given by a 95% BCa confidence interval not overlapping with zero.

#### Relationship of tree survival with functional traits, excluding or including growth performance

Estimators of selection obtained from the performance-based analysis described in Equation (1) or from the performance-mediated analysis introduced by Arnold (1983) assume that the effects of the focal traits on a fitness component are entirely mediated by the aspect(s) of performance measured. This entails the following two conditions: (a) traits do not have a direct influence on the fitness component; and (b) there are no alternative performance features through which traits exert their effects on the fitness component (Arnold, 1983; Franklin and Morrissey, 2017; for further details, see **Supplementary Methods 4**). In the present study, we relaxed condition (b) by assuming that the studied functional traits would influence mainly growth performance at the observed early stage of the life cycle, and thus their effects on other unmeasured aspects of tree performance would be less important. We thus focused on condition (a) to evaluate whether, for individuals within populations, the functional traits had direct fitness consequences, or their effects on the observed fitness component occurred only via mediation by growth performance (i.e. fitness is independent of traits, conditional on a performance measure - Franklin and Morrissey, 2017). This topic was addressed by fitting the following generalized linear FE model:


(3) $$g(E(y_{ijk}{\text{ | x}}_{ijk}))\;=\;g(\mu_{ijk}\text{) }=\;\beta_{0}\;+\;u_{j}\;+\;r_{k}\;+\;\sum_{h=1}^{p}\beta_{h}x_{h_{ijk}}\;\;$$

where *y_ijk_*, *β_0_*, *u_j_* and *r_k_* are defined as in Equation (2); the logit link function was used in *g*(.), and thus *P*(*y*_*ijk*_ = 1 | **x**_*ijk*_) was defined as described above. The linear predictor *η_ijk_* included linear terms on *p* covariates for: the functional traits alone; and the functional traits plus a given performance measure (height or diameter). In this sense, *η_ijk_* comprised firstly the three traits within the economic or hydraulic sets, and then all six traits across both sets. Incorporating height or diameter as a covariate in *η_ijk_* enables estimating the (direct) effects of functional traits on tree survival that are not mediated by growth performance. Consistent with previous models, the *x*_*h*__*ijk*___ in Equation (3) were grand-mean standardized data (expressed on a percentage scale) for the trait and performance covariates. The model parameters, statistical inference about the regression coefficients on the logit scale, and predictive ability of the fitted models, were estimated by using the methods described for Equation (2). Variance inflation factors reflecting within-population correlations between the covariates involved in the analyses were < 2, indicating a weak degree of multicollinearity.

Under Equation (1), the performance consequences of within-population variation in the traits were evaluated by considering only linear covariate terms. To keep consistency, we estimated fitness-based average directional selection gradients (see **Supplementary Methods 2**) for the focal continuous predictors in Equation (3), from modelling a fitness-phenotype function specified with only linear covariate terms in *η_ijk_*. Nevertheless, preliminary analyses showed that including nonlinear terms did not improve the model fit on the logit scale, indicating that assuming linearity of covariates and the log-odds of survival in Equation (3) would be plausible. The cluster bootstrap procedure referred to above was applied to obtain the standard errors and the 95% BCa confidence intervals of the estimated average directional selection gradients.

## Results

### Within-population correlations between the functional traits

There were five statistically significant (*p* < 0.05) within-population correlation estimates between the functional traits (**Table 1**). In the set of leaf economic traits, a significant positive correlation (0.27; *p* = 0.002) was detected between leaf thickness and leaf area. For the set of leaf hydraulic traits, the correlation between stomatal density and stomatal length was highly significant and negative (-0.58; *p* < 0.001). Across the two sets of traits, stomatal density was significantly associated with leaf thickness and leaf density, with the correlation estimates being positive (0.25; *p* = 0.004) and negative (-0.18; *p* = 0.043), respectively; a significant negative correlation (-0.22; *p* = 0.010) was also found between vein density and leaf density.

**Table 1:** Estimated within-population correlations between the functional traits, assessed in a common-garden field trial of *E. pauciflora*.

	Leaf area	Leaf density	Stomatal density	Stomatal length	Vein density
Leaf thickness	0.267 (*p* = 0.002)	-0.096 (*p* = 0.264)	0.249 (*p* = 0.004)	-0.037 (*p* = 0.670)	-0.098 (*p* = 0.257)
Leaf area		0.014 (*p* = 0.872)	0.051 (*p* = 0.552)	-0.086 (*p* = 0.315)	-0.066 (*p* = 0.439)
Leaf density			-0.175 (*p* = 0.043)	0.125 (*p* = 0.148)	-0.220 (*p* = 0.010)
Stomatal density				-0.583 (*p* < 0.001)	0.155 (*p* = 0.070)
Stomatal length					-0.039 (*p* = 0.646)

All the functional traits were assessed at age 2 years from planting. A tabulated within-population correlation was calculated from a bivariate analysis of a given pair of traits, under a (fixed-effects) linear model that was specified with populations and blocks as classification predictors. Estimation of the correlation coefficients and associated statistical inference (*p*-values within parentheses) were provided by using the MANOVA statement under the GLM procedure of SAS (SAS, 2017).

### Relationship of growth performance with functional traits

When the effects on height or diameter were modelled by using each functional trait as a single predictor, statistically significant (*p* < 0.05) ${\hat{\beta }}_{h}$ estimates were obtained for all traits except leaf area and vein density (**Table 2**). However, partialling out the within-population covariances among traits in the multiple-predictor model including all six traits showed that only leaf thickness and leaf density had statistically significant direct effects on growth performance (thinner and denser leaves were associated with faster height or diameter growth; **Table 2**). The statistically significant negative effect of stomatal density on growth performance detected in the multiple-predictor model specified for the three hydraulic traits was lost in the complete six-trait model. This indicated that the negative ${\hat{\beta }}_{h}$ estimate for stomatal density in the three-trait model reflected mainly an indirect effect arising from the significant correlations of this trait with leaf thickness and leaf density. In addition, the statistically significant positive ${\hat{\beta }}_{h}$ estimate for stomatal length modelled as a single predictor appeared to be inflated due to an indirect effect arising from the strong and negative correlation of this trait with stomatal density. In this case, the indirect effect operated via the negative influence of increasing stomatal density on growth performance which, when controlled for in the modelling of the three hydraulic traits, resulted in a small and non-significant (*p* > 0.05) ${\hat{\beta }}_{h}$ estimate for stomatal length (**Table 2**).

**Table 2:** Relationship of growth performance measured by tree height or diameter with leaf economic and hydraulic traits, assessed in a common-garden field trial of *E. pauciflora*.

Growth measure	Leaf economic traits			Leaf hydraulic traits		
	Leaf thickness	Leaf area	Leaf density	Stomatal density	Stomatal length	Vein density
*(a) Single-predictor specification*						
Height	-1.111 ± 0.255 (-1.614, -0.607) *p* < 0.001	-0.013 ± 0.107 (-0.225, 0.198) *p* = 0.900	1.585 ± 0.378 (0.837, 2.333) *p* < 0.001	-0.527 ± 0.147 (-0.818, -0.235) *p* < 0.001	0.544 ± 0.225 (0.099, 0.990) *p* = 0.017	-0.013 ± 0.132 (-0.274, 0.247) *p* = 0.919
Diameter	-1.956 ± 0.408 (-2.764, -1.148) *p* < 0.001	-0.039 ± 0.174 (-0.384, 0.305) *p* = 0.822	2.262 ± 0.624 (1.029, 3.496) *p* < 0.001	-0.971 ± 0.236 (-1.437, -0.504) *p* < 0.001	0.947 ± 0.364 (0.226, 1.668) *p* = 0.010	-0.228 ± 0.214 (-0.650, 0.194) *p* = 0.288
*(b) Multiple-predictor specification within each of the two sets of functional traits*						
Height	-1.083 ± 0.252 (-1.582, -0.585) *p* < 0.001	0.097 ± 0.100 (-0.099, 0.294) *p* = 0.329	1.425 ± 0.359 (0.715, 2.136) *p* < 0.001	-0.488 ± 0.186 (-0.855, -0.121) *p* = 0.009	0.124 ± 0.274 (-0.418, 0.665) *p* = 0.652	0.055 ± 0.129 (-0.200, 0.310) *p* = 0.669
Diameter	-1.936 ± 0.410 (-2.747, -1.125) *p* < 0.001	0.162 ± 0.162 (-0.159, 0.483) *p* = 0.320	1.978 ± 0.584 (0.822, 3.134) *p* = 0.001	-0.884 ± 0.297 (-1.471, -0.297) *p* = 0.003	0.159 ± 0.438 (-0.707, 1.026) *p* = 0.717	-0.102 ± 0.206 (-0.511, 0.306) *p* = 0.620
*(c) Multiple-predictor specification across both sets of functional traits*						
Height	-0.990 ± 0.265 (-1.515, -0.464) *p* < 0.001	0.112 ± 0.101 (-0.089, 0.313) *p* = 0.272	1.341 ± 0.371 (0.608, 2.075) *p* < 0.001	-0.201 ± 0.184 (-0.565, 0.163) *p* = 0.278	0.248 ± 0.261 (-0.268, 0.765) *p* = 0.343	0.085 ± 0.123 (-0.159, 0.330) *p* = 0.491
Diameter	-1.831 ± 0.426 (-2.674, -0.989) *p* < 0.001	0.178 ± 0.163 (-0.144, 0.500) *p* = 0.277	1.617 ± 0.594 (0.441, 2.794) *p* = 0.007	-0.393 ± 0.295 (-0.977, 0.191) *p* = 0.185	0.429 ± 0.418 (-0.398, 1.257) *p* = 0.307	-0.099 ± 0.198 (-0.491, 0.293) *p* = 0.618

The growth performance measures and the functional traits were assessed at age 2 years from planting. Modelling a growth performance measure as a function of the functional traits was pursued according to the following specifications: in *(a)*, each trait was individually modelled as a predictor variable; in *(b)*, the three traits in either economic or hydraulic set were simultaneously modelled as predictor variables; and in *(c)*, all six traits across the economic and hydraulic sets were simultaneously modelled as predictor variables. The table presents the regression coefficients estimated for the traits under a given model specification, together with the associated standard errors and 95% confidence intervals (within parentheses). Also provided are the *p*-values of statistical *t*-tests undertaken to assess whether an estimated regression coefficient differed significantly from zero. A regression coefficient estimate refers to the percentage change in relative performance (i.e. height or diameter change in % of its mean) expected from increasing a functional trait by 1%, while holding all the other explanatory variables modelled constant.

The percentage of the total variation explained (*R^2^*) in performance measures for the models fitted with single or multiple continuous predictors are given in **Supplementary Tables 2**, **3**, respectively. When compared with a base model comprising only populations and blocks as explanatory variables, the extra percentage of the total variation in growth performance (i.e. the incremental contribution to the model sums of squares relative to the corrected total sums of squares) being accounted for by the functional traits was up to 13.7%, 6.4% and 15.8% when modelling the economic, hydraulic and all six traits, respectively (**Supplementary Table 3**). In particular, leaf thickness and leaf density together had the largest relative importance for explaining the total variation in growth performance when modelling the economic traits or all six traits (i.e. the incremental contribution to the model sums of squares due to adding only both of these traits as predictors was up to 13.5%; not shown).

### Relationship of tree survival with growth performance

Based on the logistic FE model including a linear (but no quadratic) covariate term for growth performance, average directional selection gradients derived for height and diameter showed statistical support against the null hypothesis of no effect on expected relative fitness (as indicated by a 95% BCa confidence interval not overlapping with zero; **Table 3**). The fitness consequences of variation in the performance measures were both indicative of directional selection favouring individuals with higher height and diameter growth, consistent with size-dependent mortality. However, the estimated strength of directional selection on height was more than double that acting on diameter: on average, a 1% increase in height contributed to a similar change (i.e. ≈ 0.96%) in expected relative survival, whereas a 1% increase in diameter led to a change in expected relative survival of ≈ 0.44%.

**Table 3:** Relationship of tree survival with growth performance measures (height or diameter), assessed in a common-garden field trial of *E. pauciflora*.

	*(a)* Model with a linear covariate term		*(b)* Model with linear and quadratic covariate terms	
	Height	Diameter	Height	Diameter
Average directional selection gradient	0.955 ± 0.195 *(0.580, 1.320)*	0.437 ± 0.110 *(0.212, 0.639)*	0.917 ± 0.201 *(0.553, 1.308)*	0.444 ± 0.113 *(0.220, 0.657)*
Average quadratic selection gradient	–	–	-0.0158 ± 0.0142 (-0.0417, 0.0011)	-0.0057 ± 0.0045 (-0.0177, 0.0010)

The growth performance measures and tree survival (considered as a fitness component) were assessed at ages 2 and 7 years from planting, respectively. Tree survival was modelled by using a logistic model where the linear predictor included *(a)* a linear term or *(b)* linear and quadratic terms for the focal performance covariate. Estimated average directional and quadratic selection gradients, and their standard errors, are presented for a performance measure, and refer to the percentage change (on average) in expected relative survival caused by changing the covariate values expressed on a percentage scale. For example, an average directional selection gradient can be interpreted as the percentage change (on average) in expected relative survival from increasing the focal performance covariate values by 1%, conditional on the observed values of the other explanatory variables involved in the definition of the linear predictor (see **Supplementary Methods 2**). A non-parametric cluster bootstrapping procedure was pursued to obtain the standard error for a selection gradient estimate, as well as to provide the corresponding bias-corrected and accelerated (BCa) 95% confidence interval (within parentheses) (see **Supplementary Methods 3**). Statistical support against a null hypothesis being true (i.e. against the absence of an effect) is given by a 95% BCa confidence interval not overlapping with zero (indicated in Italics).

**Figure 1:**
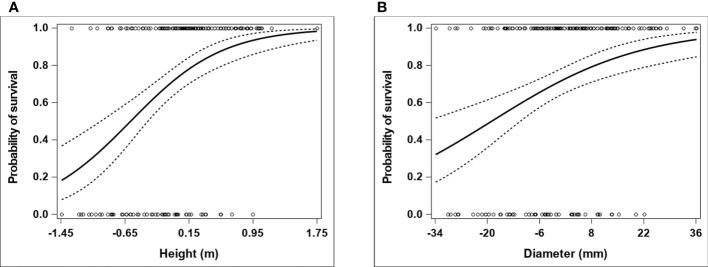
Expected probability of tree survival as a function of growth performance in *E. pauciflora*. The growth performance attributes (height and diameter) and tree survival (considered as a fitness component) were assessed at ages 2 and 7 years from planting, respectively. The plots represent fitted individual selection surfaces (and corresponding 95% confidence intervals) for **(A)** height and **(B)** diameter, based on a logistic regression model that comprised a linear term for the focal performance covariate in the linear predictor. The horizontal axis refers to absolute within-population deviations (i.e. population-mean centered observations) for a given growth performance variable. The actual observations for the fitness component (0 = dead tree; 1 = alive tree) are indicated with circles.

Adding a quadratic term for height or diameter did not significantly contribute to the fit of a logistic model that initially assumed the log-odds of survival to be linearly related to growth performance (see the estimated 95% profile penalized-likelihood confidence intervals and Tjur’s coefficient of discrimination in **Supplementary Table 4**), which is also reflected in the nearly linear trends fitted on the logit scale for the fitness-performance relationships modelling a quadratic term (**Supplementary Figure 1**). This is also consistent with the results obtained for the average quadratic selection gradients estimated on the expected fitness scale. The estimated average quadratic selection gradient was negative for either performance measure, but the associated concave downward curvature of the individual selection surface was not statistically supported, as the 95% BCa confidence interval overlapped with zero (**Table 3**). Consequently, given the indication that selection on height or diameter was mainly directional over the observed phenotypic range, the logistic model without a quadratic covariate term for growth performance leads to a more accurate estimation of the individual selection surface on the expected fitness scale. In this sense, when compared with diameter, the stronger directional selection gradient estimated for height (**Table 3**) indicates a steeper average slope of the individual selection surface for growth performance over the range spanned by the observed performance values (**Figures 1A, B**).

### Relationship of tree survival with functional traits, excluding or including growth performance

In general, the results from the analysed fitness-trait relationships did not show evidence for directional selection on the focal functional traits to be occurring via their direct influence on tree survival. Although there was some indication for fitness consequences of trait variation (e.g. leaf density) when growth performance was not modelled, there was no statistical support for a direct effect of any functional trait on tree survival when either height or diameter was included in the linear predictor in either the analyses of the three-trait sets (**Table 4**) or all the six traits (**Supplementary Table 5**). These results suggest that that the fitness component studied was independent of the focal traits, conditional on a performance measure. In contrast, statistical support for a direct effect of growth performance on tree survival was always detected in these analyses. As expected, when compared with diameter, height had a larger effect on expected relative fitness (**Table 4** and **Supplementary Table 5**) and a greater incremental contribution to the model predictive ability on the logit scale (i.e. a greater improvement in the Tjur’s coefficient of discrimination, when compared to the value of 0.27 for a model comprising only population and block effects in the linear predictor; **Supplementary Table 6**). Thus, given the results above from the performance-trait and fitness-performance relationships, the modelling of the fitness-trait relationships indicated that directional selection on the functional traits may be entirely mediated by their effects on growth performance, under the assumption that the focal traits do not influence other (unmeasured) tree performance features.

**Table 4:** Relationship of tree survival with leaf economic and hydraulic traits, and excluding or including the modelling of growth performance measures (height or diameter), assessed in a common-garden field trial of *E. pauciflora*.

	Excluding height or diameter as a predictor variable	Including height as a predictor variable	Including diameter as a predictor variable
*(a) Analyses of leaf economic traits*			
Leaf thickness	-0.548 ± 0.671 (-1.880, 0.731)	0.758 ± 0.613 (-0.396, 2.009)	0.366 ± 0.674 (-0.803, 1.848)
Leaf area	0.159 ± 0.212 (-0.258, 0.581)	0.105 ± 0.186 (-0.249, 0.488)	0.128 ± 0.205 (-0.297, 0.510)
Leaf density	1.757 ± 0.716 *(0.228, 3.023)*	0.428 ± 0.728 (-1.082, 1.690)	0.944 ± 0.737 (-0.305, 2.742)
Height	–	1.023 ± 0.255 *(0.561, 1.474)*	–
Diameter	–	–	0.424 ± 0.114 *(0.199, 0.640)*
*(b) Analyses of leaf hydraulic traits*			
Stomatal density	-0.710 ± 0.378 (-1.427, 0.048)	-0.151 ± 0.423 (-0.861, 0.826)	-0.368 ± 0.386 (-1.077, 0.434)
Stomatal length	0.218 ± 0.541 (-0.773, 1.361)	0.146 ± 0.571 (-0.898, 1.350)	0.026 ± 0.544 (-0.973, 1.187)
Vein density	-0.109 ± 0.304 (-0.671, 0.537)	-0.100 ± 0.278 (-0.628, 0.472)	-0.062 ± 0.291 (-0.609, 0.552)
Height	–	0.866 ± 0.229 *(0.478, 1.258)*	–
Diameter	–	–	0.355 ± 0.111 *(0.136, 0.565)*

The growth performance measures and the functional traits were assessed at age 2 years from planting, and tree survival (considered as a fitness component) was assessed at age 7 years. Tree survival was modelled by using a logistic model where the linear predictor included leaf economic or hydraulic traits [i.e. the *(a)* and *(b)* analyses, respectively, defined in the table] as covariates and, in both cases, it excluded or included a given growth performance variable (height or diameter). Modelling performance as a covariate in the linear predictor allows estimating the (direct) effects of functional traits on tree survival that are not mediated by growth performance (e.g. see Franklin and Morrissey, 2017). Estimated average directional selection gradients, and their standard errors, are presented for the studied covariates, and can be interpreted as the percentage change (on average) in expected relative survival from increasing the focal covariate values by 1%, conditional on the observed values of the other explanatory variables involved in the definition of the linear predictor (see **Supplementary Methods 2**). A non-parametric cluster bootstrapping procedure was pursued to obtain the standard error for a selection gradient estimate, as well as to provide the corresponding bias-corrected and accelerated (BCa) 95% confidence interval (within parentheses) (see **Supplementary Methods 3**). Statistical support against a null hypothesis being true (i.e. against the absence of an effect) is given by a 95% BCa confidence interval not overlapping with zero (indicated in Italics).

## Discussion

For the examined cohort of *E. pauciflora* individuals in the common-garden experiment, leaf thickness and leaf density had statistically significant direct effects on growth performance, but all the six leaf traits studied were found to be independent of tree survival, conditional on a growth performance measure (height or diameter). In turn, there was statistical support for a significant positive direct effect of height or diameter growth on tree survival. These results suggest that, in our study system, the effects of the focal functional traits on the observed fitness component could occur through their influence on growth performance.

### Implications for inference about directional selection on the traits

Given the above-mentioned findings, evaluating directional selection acting on the traits via their effects on performance may be pursued by performance-mediated analysis as originally described by Arnold (1983) and Arnold (2003), or by performance-based analysis as in the performance-trait relationships we have examined (for further details on these analyses, see **Supplementary Methods 4**). Under conditional independence of traits and fitness, given performance, selection inferences from either of these approaches will not be affected by a source of error due to alternative causal pathways of direct effects of traits on fitness (Franklin and Morrissey, 2017). However, although we have assumed that the effects of the functional traits on tree survival were entirely mediated by growth performance, there may be additional (unmeasured) performance features through which the traits could exert their effects on the fitness component. Consequently, strictly speaking, estimators of selection from either performance-mediated or performance-based analysis may provide a partial (rather than total) description of selection acting on the traits (i.e. pertaining only to the part of selection that is mediated by the effects of the traits on the specific performance attribute(s) measured - Arnold, 1983; Franklin and Morrissey, 2017).

Performance-mediated analysis is justified by evolutionary quantitative genetic theory, and can be applied to estimate directional and nonlinear (quadratic and correlational) selection gradients for multiple traits (Arnold, 1983; Arnold, 2003; Franklin and Morrissey, 2017). Conversely, by using performance as a substitute for fitness, performance-based analysis may not yield accurate estimates of selection gradients, which thus will limit their utility for quantitative inference about selection (Franklin and Morrissey, 2017). In the current study, performance-mediated directional selection gradients for the traits (hereafter denoted as *β_w_*) can be estimated from the product of the performance-based regression coefficients [*β_h_* in Equation (1)] shown in **Table 2** and the directional selection gradient for either performance measure given in **Table 3** (i.e. derived from the logistic model with a linear covariate term for growth performance). For selection mediated by height, the performance-based estimator of directional selection for a trait appeared to overestimate the corresponding performance-mediated directional selection gradient by 4.7% only [i.e. ( ${\hat{\beta }}_{h}$ / ${\hat{\beta }}_{w}$ ) x 100]. This indicates a relatively small magnitude of error in directional selection estimators from the performance-based analysis that used height as a fitness proxy. On the other hand, for selection mediated by diameter, the performance-based estimator of directional selection for a trait would be more than double the corresponding performance-mediated directional selection gradient. This suggests a substantial overestimation error when quantifying directional selection from the regression coefficients of the performance-based analysis that used diameter as a fitness proxy. These differences in quantifying selection between performance-based and performance-mediated analyses reflect the differences between height and diameter in regard to the directional selection gradients estimated for these performance measures. When compared with diameter, the greater effect of height on the expected probability of tree survival during the observation period between 2 and 7 years of age likely signals the importance of canopy height in avoiding light competition, as the stand becomes more closed with age (Küppers, 1994; Binkley et al., 2010).

A limitation in the comparisons described above for the performance-based and performance-mediated analyses concerns the common assumption in both approaches that the relationship of fitness with performance should be reasonably approximated with a linear function. Reflecting the cumulative distribution function of the logistic distribution, tree survival on the expected probability scale is typically nonlinearly related to a performance variable, even when a quadratic term for performance was not included in the logistic FE model. In addition, for a least-squares linear regression of a fitness response on a performance variable, Franklin and Morrissey (2017) addressed the importance of testing whether the linear function intersects the origin, in order to examine the suitability of using performance as a substitute for fitness in selection analysis. Although the logistic model is linear on the logit scale (meaning that a linear relationship is assumed between the log-odds of survival and an independent variable), this is not the scale of interest for estimating the effect of a covariate on tree survival (see **Supplementary Methods 2**), and thus it would not be meaningful to assess whether the fitness-performance function fails to intersect the origin on the logit scale. This task would be further complicated by the inclusion of blocks and populations as fixed effects in the logistic FE model, and it was not pursued in the current study.

The average curvature of the individual selection surface associated with the quadratic selection gradient estimated for either performance measure was not statistically supported (**Table 3**), hence indicating an actual monotonically increasing (sigmoid) function relating survival on the expected probability scale to a performance variable over the observed phenotypic range (**Figures 1A, B**). When accounting for a nonlinear, monotonically increasing fitness-performance relationship in a performance-mediated analysis, Franklin and Morrissey (2017) found selection gradient estimates that agreed quite closely (in particular for directional selection) with corresponding selection estimates from the Arnold (1983) approach (which assumes a linear fitness-performance function). Therefore, as concluded by Franklin and Morrissey (2017), performance-mediated analysis assuming a linear fitness-performance function may generally be quite robust, at least for inference of directional selection. This may support the results described above for the performance-mediated analysis, and (at least qualitatively) the relatively lower error in estimators of directional selection from the performance-based analysis using height as a fitness proxy in our studied system [and possibly also in the study of Costa e Silva et al. (2019), which used height data from the field trial examined here as a fitness proxy]. Further, as also indicated by Franklin and Morrissey (2017), valid qualitative within-study comparisons of selection can be provided by performance-based analysis when: it is reasonable to assume that the fitness-performance relationship is monotonic; and that the source of error due to alternative causal pathways of direct trait effects on fitness (which can influence traits differentially) is unimportant. Both of these conditions were verified in the current study, and qualitative inference of selection from the performance-based analysis indicated evidence of stronger directional selection acting on leaf thickness and leaf density - favouring thinner and denser leaves - relative to the other four functional traits studied (regardless of whether height or diameter was used as a fitness proxy; **Table 2**).

### Trait contributions to growth performance

The percentage of the total variation in early-age growth performance explained by our performance-based regression models was moderate (< 16%), indicating that other factors can be important. This is expected given the multitude of exogenous (e.g. microsite environment, inter-tree competition, browsing and disease - Costa e Silva et al., 2013; Borzak et al., 2015; Costa e Silva et al., 2017) and other endogenous factors which can affect early-age growth of eucalypts. In the latter sense, it is unlikely that maternal effects related to seed provisioning are important, as eucalypt seed is small and seed mass effects in another eucalypt species were not found to be statistically significant after two years of field growth (López et al., 2003). Nevertheless, the effects of differences among plants in resource allocation strategies (Wright et al., 2010; Costa e Silva et al., 2021) and unmeasured functional traits (Choat et al., 2018) cannot be dismissed.

Of the traits examined, performance-based directional selection was most evident for leaf thickness and density, but not detected for leaf area or the well-studied leaf economic trait - specific leaf area (SLA) (**Supplementary Table 8**) - to which the three primary economic traits studied contribute. SLA (and inverse indices such as leaf mass per area) reflects the construction costs associated with carbon acquisition, and is usually linked with leaf-level processes (e.g. photosynthetic rate, susceptibility to herbivory, leaf longevity) and plant performance (Hanley et al., 2007; Poorter et al., 2009; Sack et al., 2013; Ramírez-Valiente et al., 2014; Agrawal, 2020; Caruso et al., 2020b). SLA is generally negatively correlated with leaf density, and particularly with leaf thickness (Niinemets, 2001; Sack et al., 2013). However, leaf thickness and density reflect different facets of leaf anatomy, and they are increasingly studied separately to SLA and its inverse indices (Witkowski and Lamont, 1991; Niinemets, 2001; Poorter et al., 2009; Smith et al., 2012; Richardson et al., 2013). Such separation was important for the detection of performance-based directional selection for the economic traits in the present study. While leaf thickness and leaf density were both negatively correlated with SLA in *E. pauciflora* (**Supplementary Table 7**), they had opposing direct effects on growth performance (with increased height or diameter growth being related to thinner as well as denser leaves), leading to no detectable association between tree performance and SLA. This suggests that, at an early stage of the life cycle in our studied system, performance-based directional selection may not lead to a change in the SLA phenotypic mean, as the direct effects of leaf thickness and density on tree performance may potentially cancel those of SLA.

There are multiple mechanisms by which leaf thickness and density may independently affect tree performance. At the species-level, increased leaf thickness is usually related to increases in photosynthetically active mesophyll layers of the leaf (mainly palisade mesophyll) and longer cells within each layer (Niinemets, 2001). Such anatomical trends are also evident within eucalypt species (James and Bell, 1995; Sefton et al., 2002), including *E. pauciflora* (Körner and Cochrane, 1985). However, while increases in leaf thickness may result in more chloroplasts per unit leaf area and be favoured in high light environments, internally within the leaf, light interception as well as water and CO_2_ diffusion to the chloroplasts are enhanced by the anatomical features of thin leaves, hence facilitating the achievement of the maximum photosynthetic rate (Niinemets, 2001; Niinemets, 2007; Evans et al., 2009; Oguchi et al., 2018; Drake et al., 2019). For example, there is evidence in eucalypts that longer palisade cells decrease mesophyll conductance and create a longer CO_2_ diffusion pathway to the chloroplasts (Crous et al., 2021). Thus, thinner leaves would be expected to have enhanced internal gas diffusion to the chloroplasts which, all else being equal, would increase growth performance, as observed in the present study. This may be important for maximising photosynthetic rates and growth performance during periods when temperatures and water availability become non-limiting (Ramírez-Valiente et al., 2017).

While leaf density was weakly correlated with leaf thickness, stomatal density and vein density, its direct association with growth performance was independent of these traits (i.e. the trait covariances were partialled out in the multiple-predictor model including all six leaf traits). Increases in leaf density are mainly due to increased cell wall thickness and more tightly packed cells, which could counter the positive effects of thinner leaves on internal CO_2_ diffusion (Niinemets, 2001; Evans et al., 2009; Onoda et al., 2017). However, in eucalypts, increased leaf density can also reflect increases in leaf chemistry, including the amount of soluble proteins per unit area that may be associated with increased density of chloroplasts and photosynthetic rates (Smith et al., 2012). Thus, the positive effect on growth performance of decreased leaf thickness and increased leaf density could both signal enhanced photosynthesis. Nevertheless, there are other mechanisms favouring denser leaves in dry environments, such as their larger elastic moduli upholding water flow from drying soils (Niinemets, 2001). In addition, biotic factors may also provide independent mechanisms linking increased leaf density to growth performance. One such biotic mechanism is the thicker cell walls (associated with higher leaf density) increasing leaf toughness, and thus reducing insect herbivory (Choong, 1996; Hanley et al., 2007). Variable loss of foliage due to insect herbivory was regularly detected in the common-garden trial during the observed growing period. Eucalypts in Australia (where they are native) are subject to high levels of insect herbivory (Ohmart and Edwards, 1991), which impacts tree growth (Eyles et al., 2013). *Eucalyptus pauciflora* is no exception (Morrow, 1983; Dashiell et al., 2017), with major effects on tree performance reported (Morrow and LaMarche, 1978). Factors increasing tree stress (e.g. drought) can increase susceptibility of eucalypts to insect attack (Hanks et al., 1999; Jurskis, 2005), and potentially amplify the deleterious effects of foliage loss (e.g. low nutrients and low water; Eyles et al., 2009). Water stress, for example, will increase in the summer (Harrison, 2021), with trees experiencing competition likely more susceptible.

### Stability of the trait, growth performance and fitness associations

Over the two- to seven-year period studied, the association of tree survival with growth performance indicated that fitness benefits would arise from faster growing phenotypes. As noted previously, such size-dependent mortality is commonly reported in *ex situ* even-aged plantings of forest trees, including eucalypts (Stackpole et al., 2010; Costa e Silva et al., 2011; Nickolas et al., 2019). It is the basis of the natural thinning of planted and wild stands in which less competitive individuals fail to compete for light, water and soil nutrients (West and Borough, 1983; Peet and Christensen, 1987). In even-aged eucalypt plantings, intra-species competitive interactions among trees may commence as early as two years of age and have a genetic basis (Costa e Silva et al., 2013; Costa e Silva et al., 2017). Such competitive interactions are consistent with the present pattern of observed mortality, where the post-establishment onset of most mortality in the cohort studied commenced approximately at four years after planting (see the Materials and Methods). On the other hand, while mortality in the studied growing period tended to be higher in the smaller trees, it is recognized that the pattern of size-dependent mortality may change with age/tree size (Wright et al., 2010), with changes in direction even reported for the relationship between survival and growth (Bigler and Veblen, 2009; Lu et al., 2021). In this sense, it is hypothesised that smaller trees may be more vulnerable to mortality due to inter-tree resource competition and other biotic stresses (e.g. disease or herbivory), but larger trees more vulnerable to mortality due to abiotic stresses (e.g. wind, and water stress) (Gora and Esquivel-Muelbert, 2021). For example, greater drought damage in faster growing individuals has been reported in tree species (Martínez-Vilalta et al., 2012; Zang et al., 2014), including eucalypts (Ammitzboll et al., 2020) (but see Britton et al., 2022), and may be due to physiological trade-offs or greater depletion of soil water reserves (White et al., 2009; Wright et al., 2010; Gleason et al., 2016; Brodribb et al., 2020). In the present case, despite the above-average maximum temperatures experienced during the growing period (Costa e Silva et al., 2019), the field trial studied was not subject to a major experiment-wide abiotic stress event, and mortality gradually accumulated consistent with biotic causes, including inter-tree competition. However, empirical studies indicate that the various biotic and abiotic causes of mortality may not be independent (Camarero et al., 2016; Gleason et al., 2017; Britton et al., 2022). Thus, the changing patterns of size-dependent mortality with age may be complex and even species-specific (Bigler and Veblen, 2009; Wright et al., 2010; Britton et al., 2022). These patterns are clearly worthy of further evaluation over time in trials such as ours, especially after extreme events or as changes in climate accrue

The effects of functional traits on performance attributes and/or fitness components may also change with many factors, including tree age and canopy position (Prado-Junior et al., 2017), growth strategies (Wright et al., 2010), environment (Ramírez-Valiente et al., 2014; Siepielski et al., 2017; Ramírez-Valiente et al., 2021) and population (Costa e Silva et al., 2021; Ramírez-Valiente et al., 2021). Our results pertain to the average within-population effects of traits on a given response variable, and the applied fixed-effects model specification attempted to control for any differences in characteristics of the populations (see **Supplementary Methods 1**). However, heterogeneity in directional selection would be anticipated given the different climates from which the populations studied originate and their genetic-based differences in functional traits (Gauli et al., 2015; Costa e Silva et al., 2019; Prober et al., 2022). Indeed, in a comparison of mesic and arid adapted populations of *E. pauciflora* subject to artificial drought, Costa e Silva et al. (2021) showed statistically significant differences in directional selection gradient estimates for two of the four functional traits they studied. Nevertheless, meta-analysis indicated that directional selection tends to vary mainly in strength and less in direction among populations (Siepielski et al., 2013). Further, while evidence for frequent temporal changes in the strength and sign of directional selection gradients in natural populations may be limited (Kingsolver et al., 2012; Morrissey and Hadfield, 2012), it is likely that changes in natural selection will occur with short-term (e.g. year-to-year) climate fluctuations (Siepielski et al., 2009), particularly if rare, extreme selection events occur (Mitchell et al., 2014). For example, reduced water use efficiency in thin leaves could limit survival under extreme hot and dry conditions, imparting a direct effect on tree survival, potentially countering one of the assumptions (i.e. independence of fitness and traits, given performance) involved in performance-based and performance-mediated selection analyses. Indeed, under such circumstances, variation in leaf hydraulic traits could become more important (Choat et al., 2018).

### Implications for local adaptation

There are numerous plant economic and hydraulic traits which can affect physiological function and plant performance in the wild, and our study was focused on the association of the variation in only six commonly measured leaf traits with an early-age growth performance and a fitness component assessed in our common-garden experiment. Nevertheless, for the identified performance-based directional selection favouring thinner and denser leaves, insights into their persistence and evolutionary significance can be gauged from the associations between economic traits and home-site climate variables, as observed at the population level by Prober et al. (2022) in the same common-garden trial. In this context, statistically significant (*p* < 0.05) associations with climate were detected for leaf thickness, with populations originating from home-sites with higher average maximum temperatures tending to have thinner leaves. Thus, in terms of temperature, this trend is consistent with expectations from our performance-based analysis of directional selection at the environmental conditions of the trial site, suggesting that leaf thickness may directly contribute to local climate adaptation in the wild. In contrast to leaf thickness, the trend for performance-based directional selection to favour denser leaves was not supported by trait-climate associations [i.e. no statistically significant trait-climate associations were detected for leaf density in *E. pauciflora* by Prober et al. (2022)], arguing that the observed selective advantage of denser leaves could be more transient or site-specific.

Our studied leaf economic traits had a greater direct influence on growth performance over the period examined than the studied leaf hydraulic traits. They also exhibited more variation among populations in the study of Prober et al. (2022), signalling that they may contribute more to local adaptation of *E. pauciflora* in the wild. Nevertheless, the impact of the leaf hydraulic traits on growth performance and even tree survival may increase as competition for water increases as the studied stand ages, and climate change predictions are realised in the studied stand and the wild populations in Tasmania (increasing heat and aridity; Harrison, 2021). There are also other unmeasured hydraulic traits which may be implicated, including those related to stem hydraulic conductance and xylem vulnerability (Choat et al., 2018) and, as noted above, the leaf economic traits themselves may affect aspects of leaf hydraulics and gas exchange.

## Conclusion

In the studied *E. pauciflora* system, tree survival used as a fitness component was independent of the focal leaf economic and hydraulic traits, conditional on a performance measure (height or diameter), indicating that directional selection acting on the traits was mediated by their effects on growth performance. We provided evidence that, at an early stage of the life cycle, within-population phenotypic variation in economic traits rather than hydraulic traits directly affected growth performance, which in turn had subsequently a positive direct influence on later-age survival. Performance-based estimates of directional selection indicated that thinner and denser leaves were favoured in the hot, dry environment studied. Any association of hydraulic traits with growth performance was indirect, resulting from phenotypic correlations among the measured traits. The modelling of the fitness-performance relationship suggested that, when compared with diameter, using height as a fitness proxy may provide a relatively lower error in estimators of directional selection from performance-based analysis of selection in our studied system. While the observed selective advantage of denser leaves may be transient or site specific, the alignment of the directional selection favouring thinner leaves with predictions based on trait-climate associations for maximum temperature suggests that variation in leaf thickness may directly contribute to local climate adaptation in the species.

## Data availability statement

The datasets presented in this article are not readily available because they are currently being used in another study to be subsequently submitted for publication. Requests to access the datasets should be directed to the corresponding author for consideration.

## Author contributions

JCS conceived and conceptualised the research work undertaken in this study. BMP, SMP and GW were responsible for planning and coordinating the common-garden field trial, sampling design and trait measurements. JCS performed the data analyses, and prepared the associated document provided in Supplementary Material. JCS and BMP wrote the manuscript. All authors contributed to the manuscript revision, read, and approved the submitted version.

## Funding

The contribution of JCS to this research work was supported by *Fundação para a Ciência e a Tecnologia I.P*. (FCT), Portugal, through the *Norma Transitória* DL 57/2016/CP1382/CT0008 and UID/AGR/00239/2019. The FCT also supported this work through the funding provided to *Centro de Estudos Florestais*, Portugal (Unit Project Reference: UIDB/00239/2020). The trait data collection was co-funded by the Australian Government Department of Climate Change, Energy, the Environment and Water, and the Commonwealth Scientific and Industrial Research Organisation (CSIRO), Australia, as part of its partnership on the Biodiversity Knowledge Projects series (https://research.csiro.au/biodiversity-knowledge/), as well as the Australian Research Council Linkage (Grant LP120200380) and Industrial Transformation Training Centre (Grant IC150100004) Programs. BMP acknowledges support of the Australian Research Council Discovery grant DP190102053.

## Acknowledgments

We thank Tanya Bailey, Peter Harrison and Paul Tilyard (University of Tasmania) for their involvement in trial establishment and sample collection. We are grateful for support from Greening Australia in establishing the common garden trial, and Roderick and Kate O’Connor for supporting the trial on their property. We thank the reviewers for their time and valued comments on the manuscript.

## Conflict of interest

The authors declare that the research was conducted in the absence of any commercial or financial relationships that could be construed as a potential conflict of interest.

## Publisher’s note

All claims expressed in this article are solely those of the authors and do not necessarily represent those of their affiliated organizations, or those of the publisher, the editors and the reviewers. Any product that may be evaluated in this article, or claim that may be made by its manufacturer, is not guaranteed or endorsed by the publisher.
