# Supplementary material for: Linking leaf economic and hydraulic traits with early-age growth performance and survival of Eucalyptus pauciflora
Source: Front Plant Sci. 2022 Nov 8;13:973087. doi: 10.3389/fpls.2022.973087 (PMC9679299; doi:10.3389/fpls.2022.973087)
Supplement: Supplementary file 1 [file DataSheet_1.pdf]

Supplementary Material

Contents

1. Supplementary methods

Supplementary Methods 1: Relationship of growth performance with functional traits - linear fixed-effects and random-effects models (pages 2 to 5)

Supplementary Methods 2: Estimation of fitness-based selection gradients (pages 5 to 10)

Supplementary Methods 3: Non-parametric cluster bootstrapping for estimation of standard errors and 95% BCa confidence intervals of selection gradients (page 10)

Supplementary Methods 4: Inference of selection on traits from regression-based approaches (pages 11 to 13)

2. Supplementary figures

Supplementary Figure 1 (page 14)

3. Supplementary tables

Supplementary Table 1 (page 15)

Supplementary Table 2 (page 16)

Supplementary Table 3 (page 17)

Supplementary Table 4 (page 18)

Supplementary Table 5 (page 19)

Supplementary Table 6 (page 20)

Supplementary Table 7 (page 21)

Supplementary Table 8 (page 22)

4. Supplementary references (pages 23 to 24)

1. Supplementary methods

Supplementary Methods 1: Relationship of growth performance with functional traits - linear fixed-effects and random-effects models

Our experimental observations pertain to cross-sectional data with a two-level hierarchical structure, where (level-1) individual trees are clustered into (level-2) seed-source populations. Both fixed-effects and random-effects models can be used for the analysis of such multilevel data, in order to accommodate the dependence among observations that may occur when they are clustered into higher-level groups (e.g. Raudenbush and Bryk, 2002; Allison, 2009; Bell *et al.*, 2019). Under a fixed-effects (FE) model specification, the two-level hierarchical structure in our data can be accounted for by including dummy (indicator) variables to represent population membership, and then modelling populations as the levels of a classification predictor (as we have done). This implies that the FE model treats higher-level (population) groups as unrelated entities. In contrast, the random-effects specification of a mixed model treats higher-level groups as random draws from a common distribution, usually assumed to be the normal distribution.

Regression coefficients on between-group effects of continuous predictors cannot be estimated in the FE model, as the dummy variables coding for group identities (e.g. populations in our study) account for all the higher-level variation (Allison, 2009). Thus, the FE model uses only variation within groups, hence yielding estimates of within-group effects of continuous predictors on a response variable. By controlling out any differences in characteristics (both measured and unmeasured) of group entities, the FE model prevents the possibility of bias in the estimation of lower-level regression coefficients, that can arise from unobserved heterogeneity (omitted variables) at the higher level (Allison, 2009; Clark and Linzer, 2015; Bell *et al.*, 2019). In the current study, we favoured a FE model specification as the focus of our interest was placed on the estimation and statistical inference for within-population effects of continuous predictors (see the Materials and Methods), rather than on the estimation of population variances or the between-population effects of continuous predictors. Yet, in initial analyses of the relationship of growth performance with the functional traits, we have explored random-effects model specifications (i.e. standard and within-between linear mixed models) to compare the results obtained from them (as summarized below) with those from the linear FE model described in Equation (1) of the article.

Although the regression coefficients of continuous predictors (hereafter also referred to as "covariates") from a linear FE model will be less prone to bias due to heterogeneity in unmeasured attributes at the higher level, this may be achieved at the expense of greater sampling variability of the estimators (Allison, 2009), particularly when the number of groups and/or observations per group is not large (Clark and Linzer, 2015). By comparison, a linear mixed model may result in regression estimators on covariate effects of (level-1) individuals that are more efficient (i.e. estimated with less sampling variability), as it uses variation at both the within- and between-group data levels (Allison, 2009; Fairbrother, 2014; Clark and Linzer, 2015; Bell *et al.*, 2019). However, unbiased estimation of such regression coefficients requires that the focal covariates are uncorrelated with the random group effects (Raudenbush and Bryk, 2002; Allison, 2009; Bell and Jones, 2015; Clark and Linzer, 2015; Bell *et al.*, 2019). This restriction is not imposed under the FE model specification defined as above (e.g. Allison, 2009). In a linear mixed model, any covariance between the individual values of a covariate and the random group effects operates through the covariate's group means (Raudenbush and Bryk, 2002; page 262), which may lead to regression estimators on individual-level covariate effects being affected by bias due to omitted variables at the group level. The total error due to bias and sampling variance may thus influence statistical inference about the regression parameters from either (mixed or FE) linear model.

Using the mean square error (RMSE) of the regression coefficient estimate of a modelled covariate, Clark and Linzer (2015) compared the trade-off between efficiency and consistency (bias) of parameter estimation from a linear mixed model with a random-intercepts specification

and a linear FE model with dummy coding for group entities. Under a simulated condition (i.e. 40 groups and 5 individuals per group) that approached the number of populations and the within-population size in our study, the work of Clark and Linzer (2015) indicated that the RSME of the regression coefficient estimate from the mixed model tended to be higher than that from the FE model when the correlation between the estimated group effects for the response variable and the covariate's group means was greater than 0.4. In this context, the observed higher RSME indicated that the potential efficiency gain (i.e. reduction in sampling variability) in the regression estimator from the mixed model would no longer offset the increase in estimation bias relative to the FE model.

Standard linear mixed model

The application of a standard linear mixed model to our data was explored in initial analyses of the relationship of growth performance with the functional traits. The general model fitted for a given performance response variable (height or diameter) is described as in Equation (1), except that the population effects u_j were considered to be random draws from a normal distribution (rather than being modelled with dummy variables). We modelled covariates for the functional traits according to the following specifications: *(a)* each trait individually; and *(b)* the three traits in either economic or hydraulic set. These model descriptions also correspond to the *(a)* and *(b)* definitions provided in Table 2 of the article for the FE model. A random-intercepts specification was assumed in the mixed model, as analyses modelling only one trait at a time as a covariate [i.e. *(a)* above] did not detect a statistically significant variance (as assessed by a likelihood ratio test) associated with the heterogeneity of random slopes over populations for the focal covariate.

The variance parameters associated with the random terms - i.e. population effects (variance of level-2 random intercepts, σ_u^2) and residuals (variance of level-1 random residuals, σ_e^2) - were estimated by restricted maximum likelihood (Patterson and Thompson, 1971). Following variance component estimation, the fixed-effect parameters were estimated by generalized least-squares. In general, the distributions of the random intercepts (population effects) and residuals conformed well to the normal distribution, as indicated by the Shapiro and Wilk (1965) test of normality ($p > 0.10$), and by the visual inspection of histograms and normal quantile plots. When plotted against the fitted values, studentized deleted residuals from the examined models did not reveal residual heteroskedasticity. Statistical inference about the regression parameters (β_h) for the covariate effects of individual trees on a performance response variable was based on *t*-tests. The model-based sampling (co)variance matrix of the fixed-effect parameter estimates was corrected as described by Kenward and Roger (2009). This results in estimates of standard errors for $\hat{\beta}_h$ that account for the potential finite-sample bias and uncertainty in estimating σ_u^2 and σ_e^2 (Kenward and Roger, 2009). Based on a corrected sampling (co)variance matrix of the fixed-effect estimators, the Kenward and Roger (1997, 2009) approximation to compute degrees of freedom was applied to undertake the *t*-tests for the regression coefficients.

For a given model defined as in *(a)* or *(b)*, we used the approximation suggested by Clark and Linzer (2015) to evaluate the correlation between the covariates and the population effects (see footnote 6, page 404, of their article). The absolute value of the correlations estimated between the population means of the covariates and the population effects were ≤ 0.2 for leaf thickness, leaf density, stomatal length and vein density. However, for leaf area and stomatal density, the absolute values of the estimated correlations were ≈ 0.5 and 0.6, respectively. For these two traits, both the $\hat{\beta}_h$ estimates and the associated statistical inference changed considerably under the mixed model in comparison with corresponding results obtained from the FE model. For leaf area in the model definition *(a)* fitted to height or diameter, the *p*-values from the mixed model indicated significant regression parameters at the 10% level, but the FE model resulted in $p > 0.8$ (Table 2); in definition

(b), the mixed model led to *p*-values ≈ 0.002, whereas the FE model indicated $p \approx 0.3$ (Table 2). For stomatal density in the model definition *(a)*, the *p*-values from the mixed model were 0.018 and 0.004 for height and diameter as response variables, respectively, while the FE model resulted in highly significant ($p < 0.001$) regression parameters for the focal covariate in either case (Table 2); in definition *(b)*, the *p*-values from the mixed model were ≥ 0.15, whereas the FE model indicated significant regression parameters with *p*-values ≤ 0.009 (Table 2). For the remaining four functional traits, the conclusions from statistical inference about the regression parameters under a given model definition were not altered in the mixed model when compared with the FE model.

Within-between linear mixed model

Given the above-mentioned contrasting results between the FE model and the standard mixed model for the two covariates (leaf area and stomatal density) having the highest correlation with population effects, we further explored a linear mixed model that partitioned and explicitly modelled the within- and between-population effects of a covariate on a growth performance variable. Such specification of a linear mixed model has been recently termed as the "within-between random-effects" model (Bell *et al.*, 2018, 2019; hereafter the acronym WB is used to refer to this model), and it enables to assess whether a standard linear mixed model provides an accurate estimation of regression parameters for covariate effects of (level-1) individuals on a response variable (Raudenbush and Bryk, 2002; Allison, 2009; Bell and Jones, 2015; Bell *et al.*, 2019).

To fit the WB model to our data with a two-level hierarchical structure, the effect of hth trait on the growth performance (height or diameter) of the ith individual tree within the jth population at the kth block was partitioned into within- and between-population constituents. The former component was due to the trait deviation of a tree from its population mean ($x_{h_{ijk}}$ - $\bar{x}_{h_j}$), and the latter component was due to the trait mean of its population ($\bar{x}_{h_j}$). These constituents of the hth covariate values have separate influences on the response variable fitted by the WB model, capturing the effects associated with within- and between-population variation, as reflected in the model regression parameters β_{w_h} and β_{b_h}, respectively. In a linear WB model, all features (whether observed or not) of higher-level entities are controlled out of the estimation of lower-level regression parameters. This is because the level-1 observations based on group-mean centering (e.g. $x_{h_{ijk}}$ - $\bar{x}_{h_j}$) will not be correlated with the level-2 random effects, and thus estimates of β_{w_h} will not suffer from heterogeneity bias due to omitted variables at the group level (Goetgeluk and Vansteelandt, 2008; Fairbrother, 2014; Bell and Jones, 2015; Bell *et al.*, 2019). In this sense, estimates of β_{w_h} from a linear WB model are expected to be equivalent to corresponding estimates of regression parameters obtained for (within-group) effects of covariates under the linear FE model specification defined as above.

In addition, the WB model allows for the possibility that the within- and between-group effects of a covariate may differ. In this context, a rejection of the null hypothesis of equality of the β_{w_h} and β_{b_h} coefficients will indicate that the regression parameter estimated for the hth covariate in the standard mixed model may be biased (e.g. reflecting the heterogeneity bias mentioned above), and thereby may lack a meaningful interpretation (Raudenbush and Bryk, 2002; Allison, 2009; Bell and Jones, 2015; Bell *et al.*, 2019). Note that, under a standard linear mixed model, the within- and between-group regression coefficients of the hth covariate are constrained to be the same, leading to a regression parameter estimator on the individual-level covariate effect (β_h) that is a weighted average of the estimates of β_{w_h} and β_{b_h} from the WB model (Raudenbush and Bryk, 2002; Bell *et al.*, 2018, 2019).

The covariate specifications [*(a)* and *(b)*] and the parameter estimation procedures described before for the standard mixed model were also applied to the WB model. Following the Kenward and Roger (1997, 2009) adjustments for the correction of the model-based sampling (co)variance matrix of the fixed-effect parameter estimates and calculation of degrees of freedom, *t*-tests were pursued for statistical inference about the β_{w_h} and β_{b_h} coefficients, and their difference. As expected, for a given trait and specification of the model covariates, the parameter estimate and statistical inference for β_{w_h} in the WB model were virtually identical to the results obtained for the corresponding regression estimators in the FE model (Table 2).

In regard to statistical testing the null hypothesis of equality of β_{w_h} and β_{b_h} in the WB model, the results differed among traits. For leaf thickness, leaf density, stomatal length and vein density, the null hypothesis that β_{w_h} and β_{b_h} were the same could not be rejected ($p > 0.15$). Thus, for these four traits, a standard mixed model would be plausible for the estimation of individual-level covariate effects that would combine the variation at both levels of the hierarchical structure (yielding a weighted average of $\hat{\beta}_{w_h}$ and $\hat{\beta}_{b_h}$), hence providing regression estimators with potentially less sampling variability than $\hat{\beta}_{w_h}$ or $\hat{\beta}_{b_h}$ (Raudenbush and Bryk, 2002; Bell *et al.*, 2019). Yet, as mentioned before, using a standard mixed model was inconsequential with respect to statistical inference about the regression parameters for these four focal traits (i.e. conclusions were not altered in comparison to those drawn for the regression coefficients from the FE model, and for β_{w_h} from the WB model). However, for the remaining two traits (that had the highest estimated correlation with population effects), the null hypothesis that β_{w_h} and β_{b_h} were the same was rejected at $p < 0.05$ and $p < 0.001$ for leaf area and stomatal density, respectively. This suggested that, for either of these traits, it would have been inappropriate to combine the β_{w_h} and β_{b_h} terms for obtaining a regression estimator on the individual-level covariate effect, hence indicating that using either the WB or FE model would be more reasonable than using a standard mixed model.

In conclusion, the results obtained from the examined performance-trait relationships did not support the use of a standard linear mixed model for modelling leaf area and stomatal density as continuous predictors, and suggested that the putative efficiency gain in the regression estimators of these traits under this model may not have been sufficient to offset the bias in estimated parameter effects. Although the FE model was our choice for modelling all the functional traits (due the reasons outlined before), the WB model is more general and informative, while also enabling to unravel distinct relationships occurring at different levels of the data structure for a response variable and a set of focal covariates (for further details and discussion, see Bell *et al.*, 2019).

Supplementary Methods 2: Estimation of fitness-based selection gradients

When generalized regression analyses are used to model non-normal distributions of fitness responses, the regression coefficients entail effects on the latent scale of the response variable and cannot be quantitatively interpretable as selection gradients (Janzen and Stern, 1998; Morrissey and Sakrejda, 2013). More broadly, in generalized linear models, the estimation of marginal effects is often a useful approach to evaluate and interpret the outcomes of changes in continuous predictor variables on the observed scale of the response variable (Breen *et al.*, 2018). This was the case of the logistic fixed-effects (FE) models applied in our study, as it was desirable to express the effects of performance measures and functional traits on the expected probability scale (rather than log-odds or odds ratios) of tree survival. In this context, the marginal effect for a continuous predictor at

an observation point measures the instantaneous effect on the expected probability scale of tree survival due to a change in the focal covariate, conditional on the observed values of the other explanatory variables that define the linear predictor of the logistic FE model. Therefore, the estimation of a marginal effect involves the application of a partial derivative of the conditional expectation of tree survival with respect to the focal covariate at an individual observation point.

When modelling the relationship of tree survival with growth performance, individual first partial derivatives were calculated to obtain an average directional selection gradient for performance (height or diameter) under a logistic FE model that included a linear term [as in Equation (2)] or both linear and quadratic terms for the performance variable. In addition, individual second partial derivatives were computed to obtain an average quadratic selection gradient for performance, only when the model comprised both linear and quadratic terms for the performance variable. When modelling the relationship of tree survival with functional traits, and excluding or including growth performance, individual first partial derivatives were also calculated to obtain average directional selection gradients for the focal covariates, under the model specified as in Equation (3). Two methods were compared for the calculation of first and second partial derivatives: an approximation based on numerical methods entailing (second-order) central finite differences; and using analytical expressions based on differentiation rules. As described below, these methods resulted in identical estimates for average derivatives (and corresponding average selection gradients). The details on the estimation of average (fitness-based) selection gradients using either method of derivative calculation are provided as follows.

Selection gradient estimation based on numerical approximation of the partial derivatives

Rather than using an analytical definition based on differentiation rules, the partial derivative of a response function with respect to a continuous predictor at an individual observation point can be approximated by numerical methods (e.g. Morrissey and Sakrejda, 2013; Franklin and Morrissey, 2017; Williams, 2021). We have applied (second-order) central finite differences to numerically approximate first and second partial derivatives with respect to a focal covariate, and evaluated on the expected probability scale of tree survival at each individual within a population. These numerical derivatives were averaged over all the examined individuals and subsequently expressed on a expected relative fitness scale, in order to provide a fitness-based estimate of an average selection gradient for the focal covariate. In this sense, a selection gradient estimate refers to a summary measure of the strength and form of selection occurring within populations across the distributional range of *E. pauciflora* in Tasmania. The following steps were pursued.

(i) Fit a given logistic FE model to the binary response (tree survival), and estimate the model parameters on the logit scale (log-odds of survival).

(ii) Obtain the expected probability of survival at every observation, conditional on the observed values of all the explanatory variables modelled (in our study, including also populations and blocks as classification predictors). This refers to $P(y_{ijk}=1|\mathbf{x}_{ijk})$ for the ith individual within the jth population at the kth replicate, as defined in the Materials and Methods, and the corresponding expected value [hereafter denoted as $\hat{p}(y\,|\,x)_{ijk}$] from the fitted logistic FE model is given by:

$$\hat{p}(y\,|\,x)_{ijk} = \frac{exp(\mathbf{x}_{ijk}\hat{\boldsymbol{\beta}})}{1+exp(\mathbf{x}_{ijk}\hat{\boldsymbol{\beta}})} \quad \text{(S2\_1)}$$

where $\mathbf{x}_{ijk}$ is a row vector for an individual from the design matrix $\mathbf{X}$ of all the explanatory variables in the linear predictor (η_{ijk}), *exp* denotes the exponential function, and $\hat{\boldsymbol{\beta}}$ is a column vector of coefficient estimates on the logit scale pertaining to the fixed-effect parameters modelled.

(iii) For a given focal covariate x_h, change its values by a small amount Δ below and above an observation of x_h (i.e. $x_{h_{ijk}}$ - Δ and $x_{h_{ijk}}$ + Δ, for an individual), while holding all the other explanatory variables modelled at their observed values.

(iv) Using Equation (S2_1) with the estimated parameters from the fitted logistic FE model, predict the probability of survival at every individual after changing the x_h observations by either (- or +) Δ value. Thus, two predictions of the probability of survival were obtained for a given individual: one referring to observed values of x_h changed to $x_{h_{ijk}}$ - Δ, and another pertaining to observed values of x_h changed to $x_{h_{ijk}}$ + Δ; we denote these predictions as $\hat{p}(y \mid x, x_h - \Delta)_{ijk}$ and $\hat{p}(y \mid x, x_h + \Delta)_{ijk}$, respectively - in this notation, x refers to all explanatory variables in the model except x_h, with their values kept as originally observed, as mentioned in (iii).

(v) At each individual (i.e. at the *ijk* observation point), numerically evaluate the partial derivatives of the conditional expectation of survival with respect to the focal covariate x_h, using (second-order) central finite difference approximations to the first and second partial derivatives as follows:

$$\frac{\partial E(y_{ijk} \mid \mathbf{x}_{ijk})}{\partial_{x_h}} \approx \frac{\hat{p}(y \mid x, x_h + \Delta)_{ijk} \ - \ \hat{p}(y \mid x, x_h - \Delta)_{ijk}}{2\Delta} \tag{S2\_2}$$

$$\frac{\partial^2 E(y_{ijk} \mid \mathbf{x}_{ijk})}{\partial_{x_h^2}} \approx \frac{\hat{p}(y \mid x, x_h + \Delta)_{ijk} \ - \ 2\hat{p}(y \mid x)_{ijk} \ + \ \hat{p}(y \mid x, x_h - \Delta)_{ijk}}{\Delta^2} \tag{S2\_3}$$

with the second derivative in Equation (S2_3) being calculated for a performance measure when modelling the relationship of survival with growth performance, and under a logistic FE model that included linear and quadratic terms for the performance variable (as referred above, and also in the Materials and Methods). It is also important to note that, for the logistic FE model that comprised linear and quadratic terms for growth performance, squared values of the performance covariate were obtained after population-mean centering the x_h values as described in Materials and Methods [see also below in regard to Equations (S2_5) and (S2_6)], which will be reflected in the model predictions of the probability of survival included in Equations (S2_2) and (S2_3).

(vi) Average the first and second partial derivatives calculated in Equations (S2_2) and (S2_3), respectively, over all the examined individuals. Averaging the individual derivatives will reflect the observed distribution of the focal covariate x_h, yielding a quantity that summarizes an average contribution of the covariate on the expected probability scale of tree survival, conditional on the observed values of the other explanatory variables (in our study, including also population and block effects) involved in the definition of the linear predictor of the logistic FE model.

(vii) As fitness-based selection gradients are usually provided in terms of relative fitness rather than absolute fitness (Lande and Arnold, 1983), express an average derivative on a expected relative fitness scale to estimate an average selection gradient for x_h. This was undertaken via the division of an average derivative by the mean of the expected survival probabilities of all individuals, which was estimated by $\frac{1}{n}\sum_{i=1}^{n}\hat{p}(y \mid x)_{ijk}$ (e.g. Morrissey and Sakrejda, 2013) under a given definition of the linear predictor η_{ijk} of the logistic FE model. Thus, estimated (fitness-based) average directional and quadratic selection gradients (the latter for a performance measure only) pertain to the average of individual first and second numerical derivatives, respectively, expressed on the expected relative survival scale.

Reflecting the cumulative distribution function of the logistic distribution, tree survival on the expected probability scale will typically be nonlinearly related to a focal covariate x_h, even when only linear terms are included in the logistic FE model for the covariates. Thus, the marginal effect of x_h depends on the covariate's own value at the observation point (i.e. marginal effects vary over the observed range of x_h), and strictly refers to an instantaneous effect on $P(y_{ijk} = 1 \mid \mathbf{x}_{ijk})$ for a small change Δ in x_h [which, depending on how x_h is scaled, may or may not approach the effect on $P(y_{ijk} = 1 \mid \mathbf{x}_{ijk})$ for a one unit change in x_h; see below]. This instantaneous effect is consistent with the notion of a partial derivative, with the corresponding numerical approximation converging to an exact derivative as Δ approaches zero (Williams, 2021).

The choice of the Δ value for calculating numerical derivatives will depend on the units (scale) of x_h and, in this context, Cameron and Trivedi (2010) suggested using the standard deviation of the focal covariate divided by 1000. This led to Δ values ranging from 0.01 to 0.04 for the studied continuous predictors (performance measures and functional traits), using covariate observations that were previously population-mean centered, then grand-mean standardized and finally expressed on a percentage scale. We have chosen the smallest of these Δ values (i.e. 0.01) to compute the numerical derivatives in Equations (S2_2) and (S2_3). This yielded average values of the numerical derivatives for the studied covariates (and corresponding average selection gradients) that were identical to the estimates obtained from the first and second partial derivatives calculated analytically (see their description below).

In addition, the scale of x_h will also determine whether or not the covariate's marginal effect at an observation approaches the effect on $P(y_{ijk} = 1 \mid \mathbf{x}_{ijk})$ for a one unit change in x_h (Williams, 2021). As mentioned above, the observations of the studied continuous predictors were expressed on a percentage scale. Consequently, for a given definition of the linear predictor η_{ijk} of the logistic FE model, an estimated average selection gradient refers to the change (on average) in expected relative survival caused by changing the focal covariate values expressed on a percentage scale. In this sense, we also computed the numerical derivatives in Equations (S2_2) and (S2_3) by using the Δ = 1 to reflect a change of 1% in x_h. This resulted in average estimates of the numerical derivatives for the target covariates that were similar to those obtained from Δ = 0.01. Thus, for example, an average directional selection gradient can be interpreted as referring to the change (on average) in expected relative survival from increasing the focal covariate values by 1%, conditional on the observed values of the other explanatory variables involved in the definition of the linear predictor. Note that the results provided in Tables 3, 4, and Supplementary Table 5, pertain to estimated average changes in expected relative survival that were also converted to percentages.

Finally, the above-mentioned nonlinear relationship between tree survival on the expected probability scale and a focal covariate also implies that the marginal effects of x_h across its observed range depend on the observed values of all the other explanatory variables modelled. Consequently, computing the numerical derivatives in Equations (S2_2) and (S2_3) with respect to x_h required conditioning on all the other explanatory variables included in the logistic FE model, which we have done by leaving the values of these variables as they were originally observed (rather than, for example, holding other modelled covariates at fixed values, such as at their means).

Selection gradient estimation based on partial derivatives calculated analytically

The analytical calculation of a partial derivative of a regression function with respect to a given continuous predictor involves the definition of the partial derivative using differentiation rules. Subsequently to the estimation steps (i) and (ii) described above, we computed analytical first and second partial derivatives with respect to a focal covariate x_h on the expected probability scale of tree survival. The following analytical expressions obtained from differentiation rules were used to calculate first and second partial derivatives for each individual within a population.

When only linear terms were used in the logistic FE model for the studied covariates [i.e. either performance measure in Equation (2); and functional traits, excluding or including a performance measure, in Equation (3)], the first partial derivative of the conditional expectation of survival with respect to the focal covariate x_h at each observation point was computed by:

$$\frac{\partial E(y_{ijk} \mid \mathbf{x}_{ijk})}{\partial_{x_h}} = \hat{p}(y \mid x)_{ijk} \cdot (1 - \hat{p}(y \mid x)_{ijk}) \cdot \hat{\beta}_h \qquad \text{(S2\_4)}$$

where $\hat{p}(y \mid x)_{ijk}$ was estimated as defined in Equation (S2_1), and $\hat{\beta}_h$ is the estimated regression coefficient on the logit scale for the linear term of x_h. Equation (S2_4) was proposed by Janzen and Stern (1998) for estimation of average directional selection gradients from logistic regression models including only linear terms for the focal covariates.

When the fitted logistic FE model had linear and quadratic terms for a covariate [i.e. either performance measure, after extending Equation (2) to include a quadratic term for performance], the first and second partial derivatives of the conditional expectation of survival with respect to the focal covariate x_h at each observation point were calculated by:

$$\frac{\partial E(y_{ijk} \mid \mathbf{x}_{ijk})}{\partial_{x_h}} = \hat{p}(y \mid x)_{ijk} \cdot (1 - \hat{p}(y \mid x)_{ijk}) \cdot (\hat{\beta}_{h_1} + 2\hat{\beta}_{h_2} x_{h_{ijk}}) \qquad \text{(S2\_5)}$$

$$\frac{\partial^2 E(y_{ijk} \mid \mathbf{x}_{ijk})}{\partial_{x_h^2}} = \hat{p}(y \mid x)_{ijk} \cdot (1 - \hat{p}(y \mid x)_{ijk}) \cdot \left[[(\hat{\beta}_{h_1} + 2\hat{\beta}_{h_2} x_{h_{ijk}})^2 \cdot (1 - 2\hat{p}(y \mid x)_{ijk})] + 2\hat{\beta}_{h_2} \right] \qquad \text{(S2\_6)}$$

where $\hat{p}(y \mid x)_{ijk}$ was estimated as defined before; $\hat{\beta}_{h_1}$ and $\hat{\beta}_{h_2}$ are the estimated regression coefficients on the logit scale for the linear and quadratic terms, respectively, of x_h; and $x_{h_{ijk}}$ is the *centered* observation of x_h for the ijk^{th} individual. The centered $x_{h_{ijk}}$ data in Equations (S2_5) and (S2_6) stems from fact that it was required when Equation (2) in the survival-performance

relationship was extended to model a quadratic term for the performance covariate. In this sense, the x_{ijk} data were population-mean centered (by subtracting the $\bar{x}_j$ mean from all observations belonging to the jth population) as described in the Materials and Methods, in order to avoid multicollinearity and to obtain covariate values for the estimation of a within-population, nonlinear effect of growth performance on the logit scale. Otherwise, for data without population structure, the observations could be centered by using the grand mean of the focal covariate.

As for the numerical derivatives, the analytical derivatives with respect to a focal covariate x_h at an observation point were computed while holding all the other explanatory variables modelled at their observed values. These individual first and second analytical derivatives were averaged over all the examined individuals, and then expressed on the expected relative survival scale [as described in step (vii) above] to provide estimates of (fitness-based) average directional and quadratic selection gradients (the latter for a performance measure only), respectively. As mentioned before, these estimates were identical to those obtained by using numerical derivatives.

Supplementary Methods 3: Non-parametric cluster bootstrapping for estimation of standard errors and 95% BCa confidence intervals of selection gradients

A non-parametric cluster bootstrapping procedure was performed to obtain the standard errors for the fitness-based selection gradient estimates, and to provide the corresponding 95% confidence intervals. Cluster bootstrapping is a resampling method that can be used to reflect the two-level hierarchical structure that is present in cross-sectional data with individuals clustered into groups. In this context, resampling is undertaken at the group level rather than at the individual level, such that the within-group composition is kept for a group randomly drawn into a bootstrap sample (Davison and Hinkley, 1997). The cluster bootstrapping may be supplemented by an additional, second-stage resampling of individuals within resampled groups. However, such two-step procedure was not applied in the current study since the within-population sizes in the original data were not large, in which case the second-stage resampling could give inaccurate bootstrap results and lead to numerical problems (Van der Leeden *et al.*, 2008).

Populations were sampled with replacement by using a balanced bootstrap method (Gleason, 1988; Davison and Hinkley, 1997); 9999 bootstrap samples were generated, with the balanced bootstrap resulting in each population being selected 9999 times over all bootstrap replications. Unique identifiers were given to resampled populations in each bootstrap sample, and the data were grand mean-standardized (and also initially population-mean centered to model the quadratic term for the performance covariate in the survival-performance relationship; see Materials and Methods) by using estimated means from each bootstrap sample. Monitoring the convergence of the logistic fixed-effects model detected a small percentage of bootstrap samples where convergence was not attained. This was observed when both linear and quadratic terms were modelled for height in the survival-performance relationship (referring to results shown in Table 3), and when the linear predictor comprised height together with economic or hydraulic traits in the relationship of survival with functional traits including growth performance (pertaining to results given in Table 4). These few bootstrap samples where the model failed to converge were discarded from further analyses.

Fitness-based selection gradients for the focal continuous predictor variables were estimated in each bootstrap sample as detailed in Supplementary Methods 2, and the standard error of a selection gradient was obtained from the standard deviation of its bootstrapped estimates. Bias-corrected and accelerated (BCa) 95% confidence intervals were computed as described by Efron and Tibshirani (1993). In this context, for a given focal covariate, the acceleration factor was calculated by using jackknife values of the selection gradient, which were obtained by removing each population in turn and then re-estimating the selection gradient with that population omitted. Statistical support against a null hypothesis being true (i.e. against the absence of an effect associated with a selection gradient) was given by a 95% BCa confidence interval not overlapping with zero.

Supplementary Methods 4: Inference of selection on traits from regression-based approaches

Lande and Arnold (1983) established a regression-based framework for quantitative inference about natural selection, founded on the relationship between relative fitness (or a relative fitness component referring to, for example, age-specific survival or fecundity) and a set of focal traits comprising a multivariate phenotype. The regression coefficients from this approach enable the estimation of *fitness-based* selection gradients that have a known link to evolutionary quantitative genetic theory (Lande, 1979; Lande and Arnold; 1983; Walsh and Lynch, 2018). When fitness and traits cannot be assessed on the same individuals, inference of selection acting on traits may be drawn from regression parameters obtained by *performance-mediated* or *performance-based* analyses (Arnold 1983, 2003; Franklin and Morrissey, 2017). As described below, either of these approaches involves the modelling of attributes that affect organismal performance (such as growth measures), which are expected to be related to fitness or fitness components.

Performance-mediated analysis

Arnold (1983) used path analytical methods to introduce the concept of performance-mediated directional selection gradients for a set of focal traits. In this framework, a performance attribute lies in between fitness and the traits, and a linear (directional) selection gradient is obtained by combining regression parameters estimated from the modelling of performance-trait and fitness-performance relationships. For the simple case of a single trait z influencing a single performance measure f, the pathway from z to relative fitness w can be partitioned into two parts comprising the regression parameters from the linear regression of performance on the trait and from the linear regression of relative fitness on performance, hereafter denoted as β_{fz} and β_{wf}, respectively. The β_{fz} and β_{wf} parameters were termed by Arnold (1983) as "performance gradient" for the trait and "fitness gradient" for performance, respectively. The performance-mediated directional selection gradient for z (β_{wz}) can thus be obtained via the product $\beta_{wz} = \beta_{fz}\beta_{wf}$ (Arnold, 1983). This approach can be extended to estimate partial regression parameters referring to directional and non-linear (quadratic and correlational) selection gradients for multiple traits, and also to include more than one pathway linking traits to fitness (e.g. traits directly influencing two or more distinct performance measures, which in turn directly affect fitness) (Arnold, 1983, 2003; Franklin and Morrissey, 2017).

Performance-mediated analysis assumes a causal structure where selection on the focal traits (and thus their effects on fitness or on a fitness component) is entirely mediated by the aspect(s) of performance measured. This entails the following two conditions: (i) traits do not have a direct influence on fitness; and (ii) there are no alternative performance features through which the traits exert their effects on fitness (Arnold, 1983; Franklin and Morrissey, 2017). Condition (i) implies that fitness is independent of the traits, conditional on the performance attribute(s) measured. Condition (ii) implies that the traits do not influence other (unobserved) aspects of organismal performance beyond the performance measure(s) modelled. However, when there are alternative pathways that include traits affecting fitness directly and/or influencing unmeasured performance features, performance-mediated selection gradients can still provide a partial (rather than total) description of selection acting on traits [i.e. referring only to the part of selection that is mediated by the specific performance attribute(s) measured; Arnold, 1983; Franklin and Morrissey, 2017]. In addition, performance-mediated analysis assumes a linear fitness-performance relationship (Arnold, 1983, 2003). Yet, Franklin and Morrissey (2017) reported that selection gradients estimated by a performance-mediated analysis that accounted for a non-linear, monotonically increasing fitness-performance relationship agreed quite closely (in particular for directional selection) with corresponding selection estimates from Arnold's (1983) approach (which assumes a linear fitness-performance function). In any case, both fitness-based and performance-mediated selection gradients may not reflect total direct selection on a set of traits, as estimates may be obtained via

observed trait effects on a fitness component (which usually refers to an event of selection, rather than to lifetime fitness), and may also include potential indirect effects of selection on unmeasured characters that are correlated with the observed traits (Arnold, 1983; Lande and Arnold, 1983).

Arnold's (1983) performance-mediated selection analysis has a justification in evolutionary quantitative genetic theory, and is especially helpful when it may be unfeasible to measure fitness and traits on the same organisms, in which case performance-trait and fitness-performance relationships could use data from different individuals (e.g. separate studies within a species). Regardless of this motivation, assessing both the influence of traits on performance and the effect of performance on fitness will still be important to gain a broad view of selection and adaptation (Arnold, 1983). Furthermore, using information from both performance-trait and fitness-performance relationships will be needed to evaluate the conditions under which it is plausible that selection can be quantitatively inferred by substituting performance attributes for fitness or fitness components under a performance-based analysis. These conditions have been addressed in detail by Franklin and Morrissey (2017), and will be summarized below.

Performance-based analysis

In studies where fitness cannot be determined, inference of selection acting on traits has also been drawn from examining the relationship between a fitness proxy and the multivariate phenotype. In this context, fitness proxies have usually entailed performance attributes that are expected to be positively related to fitness or fitness components (see Franklin and Morrissey, 2017, for a review of studies using performance measures as substitutes for fitness). In essence, such performance-based analysis pertains only to one of the parts involved in Arnold's (1983) performance-mediated analysis (i.e. the performance-trait relationship). However, in contrast to fitness components, fitness proxies based on organismal performance at a given stage of the life cycle cannot be assumed to represent the demographic contribution of individuals to future generations. Consequently, as underlined by Franklin and Morrissey (2017), performance-based estimators of selection obtained from multiple regression of a performance measure (commonly expressed on a relative scale, via the division of the individual observations by the mean value) on the multivariate phenotype may not be reflective of fitness-based (or performance-mediated) selection gradients and their inherent evolutionary quantitative genetic interpretation.

Sources of the total error associated with selection estimators obtained by using performance measures in place of fitness may be due to: alternative pathways via which traits affect fitness; and the nature of the fitness-performance relationship. Thus, along with the conditions (i) and (ii) defined above, accurate estimation of selection gradients from performance-based regression coefficients requires: a linear fitness-performance relationship (as in the performance-mediated analysis); and a small proportional error introduced by the linear fitness-performance relationship on the performance-based estimators of selection (Franklin and Morrissey, 2017). Under a linear regression model, Franklin and Morrissey (2017) demonstrated that the latter source of error may arise from the intercept of the fitness-performance relationship not passing through the origin, and it depends on the value of the intercept relative to the slope.

As shown by Franklin and Morrissey (2017) for the part of selection mediated by organismal performance, the proportional error introduced by a linear fitness-performance relationship on the selection estimate for a trait from performance-based analysis can be ascertained by comparing this estimate with the corresponding selection gradient calculated from the performance-mediated analysis. Considering directional selection on a trait z as an example, and using a performance measure expressed on a relative (mean-standardized) scale, the performance-mediated directional selection gradient (β_{wz}) for z can be obtained by $\beta_{wz} = \beta_{fz}\beta_{wf}$ (as described above), where: β_{fz} = performance-based gradient for the trait (from the regression of relative performance f on z); and β_{wf} = fitness-based gradient for performance (from the regression of relative fitness w on relative performance f). The proportion by which β_{fz} underestimates or overestimates β_{wz} can thus be given

by $\beta_{fz} / \beta_{wz} = 1/\beta_{wf}$ (Franklin and Morrissey, 2017), indicating that the proportional error will be small when $\beta_{wf} \approx 1$. Yet, as indicated by Franklin and Morrissey (2017), performance-based estimators of selection (and including both directional and non-linear terms) for the focal traits will be influenced equally by the proportional error introduced by the fitness-performance relationship. This suggests that valid qualitative within-study comparisons of selection may still be provided by performance-based analysis when it is reasonable to assume that the fitness-performance relationship is monotonic, and that the source of error due to alternative causal pathways of trait effects on fitness (which can influence traits differentially) is unimportant (Franklin and Morrissey, 2017). Such qualitative inference of selection can be useful despite the putative limited justification that performance-based estimators of selection may have in evolutionary quantitative genetic theory.

2. Supplementary figures

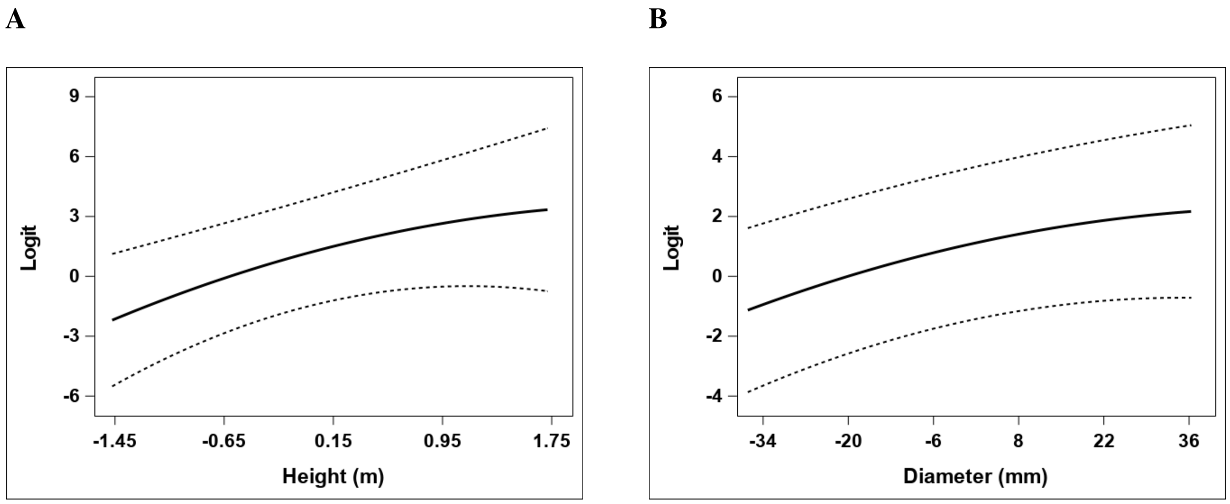

Supplementary Figure 1 | Relationship between tree survival (scored as a binary response) and a growth performance variable (height or diameter) based on a logistic fixed-effects model where the linear predictor included linear and quadratic terms for the focal performance covariate. The plots show fitted functions (and corresponding 95% confidence intervals) on the logit (log-odds of survival) scale for (**A**) height and (**B**) diameter. The horizontal axis refers to absolute within-population deviations (i.e. population-mean centered observations) for a growth performance variable (note that using alternatively relative, grand-mean standardized, within-population deviations does not alter the shapes of the fitted functions illustrated in the figure). The nearly linear trends depicted on the logit scale reflect the results given in Supplementary Table 4, which indicated no statistical support (i.e. 95% profile penalized-likelihood confidence intervals overlapping with zero) for the presence of a significant quadratic effect of either performance measure on the log-odds of survival.

3. Supplementary tables

Supplementary Table 1 | Estimated means (with the range of observations within parentheses) and standard deviations for leaf economic traits, leaf hydraulic traits, and growth performance measures, assessed in a common-garden field trial of *E. pauciflora* at age 2 years from planting.

	Mean	Standard deviation (raw observations)	Standard deviation (population-mean centered observations)
Leaf economic traits			
Leaf thickness (mm)	0.512 (0.405 - 0.766)	0.054	0.043
Leaf area (cm^2)	21.35 (7.41 - 46.67)	6.57	4.44
Leaf density (mg/mm^3)	0.420 (0.324 - 0.507)	0.030	0.026
Leaf hydraulic traits			
Stomatal density ($number/mm^2$)	138.84 (84.99 - 226.47)	24.56	19.96
Stomatal length (µm)	35.57 (25.97 - 48.26)	3.94	3.53
Vein density (mm/mm^2)	10.47 (6.20 - 17.32)	2.04	1.81
Growth performance measures			
Height (m)	2.24 (0.70 - 4.80)	0.736	0.599
Diameter (mm)	35.39 (2.83 - 81.57)	19.08	15.83

Supplementary Table 2 | Percentage of the total variation explained (i.e. R^2) in growth performance measures (height or diameter) for the linear models including a functional trait as a single continuous predictor variable.

Growth measure	Base model [a)]	Leaf thickness	Leaf area	Leaf density	Stomatal density	Stomatal length	Vein density
Height	38.6%	46.1%	38.6%	45.6%	43.5%	40.8%	38.6%
Diameter	39.5%	48.0%	39.5%	44.5%	45.8%	41.9%	40.0%

The base model refers to a specification comprising populations and blocks as fixed-effects parameters, but no predictors for the functional traits; the corresponding R^2 values are given as a reference for comparison. The other R^2 values pertain to the extension of the base model to include a functional trait as a single continuous predictor variable (described in Table 2 of the article as "Single-predictor specification"). When compared with the R^2 values for the base model, the R^2 values for the other models reflect the additional percentage of the total variation in a growth performance measure (i.e. the incremental contribution to the model sums of squares relative to the corrected total sums of squares) that was accounted for by the functional trait being modelled as a predictor variable.

[a)] Compared with a model specification including only block effects, incorporating population effects in the base model added 33.7% and 31.3% to the percentage of the total variation explained in height and diameter, respectively. Thus, population effects had the largest incremental contribution to the sums of squares (and thus to the overall R^2) of the base model, consistent with the high statistical significance (i.e. p = 0.001 and p = 0.002 for height and diameter, respectively) detected for the population term under this model.

Supplementary Table 3 | Percentage of the total variation explained (i.e. R^2) in growth performance measures (height or diameter) for the linear models including multiple continuous predictor variables within or across the economic and hydraulic sets of functional traits.

Growth measure	Base model [a)]	Model including the three economic traits	Model including the three hydraulic traits	Model including all the six functional traits
Height	38.6%	52.3%	43.5%	53.9%
Diameter	39.5%	52.6%	45.9%	55.3%

The base model refers to a specification comprising populations and blocks as fixed-effects parameters, but no predictors for the functional traits; the corresponding R^2 values are given as a reference for comparison. The other R^2 values pertain to the extension of the base model to include functional traits as continuous predictor variables, and entailing the multiple trait modelling of: the three functional traits in either economic or hydraulic set (described in Table 2 of the article as "Multiple-predictor specification within each of the two sets of functional traits"); or all the six functional traits across both sets (described in Table 2 of the article as "Multiple-predictor specification across both sets of functional traits"). When compared with the R^2 values for the base model, the R^2 values for the other models reflect the additional percentage of the total variation in a growth performance measure (i.e. the incremental contribution to the model sums of squares relative to the corrected total sums of squares) that was accounted for by the functional traits being modelled as predictor variables.

[a)] Compared with a model specification including only block effects, incorporating population effects in the base model added 33.7% and 31.3% to the percentage of the total variation explained in height and diameter, respectively. Thus, population effects had the largest incremental contribution to the sums of squares (and thus to the overall R^2) of the base model, consistent with the high statistical significance (i.e. p = 0.001 and p = 0.002 for height and diameter, respectively) detected for the population term under this model.

Supplementary Table 4 | Relationship of tree survival with growth performance measures (height or diameter), assessed in a common-garden field trial of *E. pauciflora*. The results refer to estimated coefficients of regression parameters on the logit (log-odds) scale.

	(a) Model with a linear covariate term		*(b)* Model with linear and quadratic covariate terms	
	Height	Diameter	Height	Diameter
Tjur's coefficient of discrimination	0.468	0.379	0.470	0.384
Survival regressed on absolute values of performance				
Linear term	2.088<br>*(1.266, 3.119)*	0.053<br>*(0.027, 0.083)*	1.831<br>*(1.043, 2.909)*	0.050<br>*(0.025, 0.078)*
Quadratic term	-	-	-0.697<br>(-2.099, 0.786)	-0.001<br>(-0.003, 0.001)
Survival regressed on percentage values of performance				
Linear term	0.0468<br>*(0.0284, 0.0699)*	0.0188<br>*(0.0096, 0.0293)*	0.0410<br>*(0.0234, 0.0651)*	0.0175<br>*(0.0088, 0.0276)*
Quadratic term	-	-	-0.0004<br>(-0.0011, 0.0004)	-0.0001<br>(-0.0003, 0.0001)

The growth performance measures and tree survival (considered as a fitness component) were assessed at ages 2 and 7 years from planting, respectively. Tree survival was scored as a binary response, which was modelled with a (fixed-effects) generalized linear model using the logit link function to relate the conditional expectation of the response variable to a linear predictor. Besides including populations and blocks as classification predictor variables, the linear predictor also comprised *(a)* a linear term or *(b)* linear and quadratic terms for the performance covariate (height or diameter). The results are provided for survival regressed on either absolute (with measurement scales in m and mm for height and diameter, respectively) or relative (i.e. percentage values following mean-standardization by using the grand means of either performance measure) values of growth performance. Note that all of the results presented for the quadratic term are based on population-mean centered observations of the performance covariate (see the Materials and Methods). In particular, under the *(a)* model definition, the tabulated coefficient of a regression parameter refers to the change in the log-odds of the response (i.e. the log-odds that survival equals one) for a one unit increase (e.g. 1m or 1% change in height) in the focal performance measure, while holding the other explanatory variables modelled constant. The estimation of the model parameters was based on the bias-reducing penalized maximum likelihood method described by Firth (1993); 95% profile penalized-likelihood confidence intervals are provided within parentheses. Statistical support against a null hypothesis being true (i.e. against the absence of an effect for a linear or a quadratic term of performance on the log-odds of survival) is given by a 95% confidence interval not overlapping with zero (indicated in Italics). The Tjur's coefficient of discrimination (Tjur, 2009) is presented as a measure of the predictive ability of the model; as a reference value, a logistic model comprising only populations and blocks as fixed-effects parameters in the linear predictor resulted in a Tjur's coefficient of discrimination of 0.269.

Adding a quadratic term for height or diameter did not significantly contribute to the fit of a logistic fixed-effects model that initially assumed the log-odds of survival to be linearly related to growth performance. The 95% profile penalized-likelihood confidence intervals for the quadratic regression parameters on logit scale overlapped with zero, and there were minor improvements in the Tjur's coefficient of discrimination when extending the model to include the quadratic term for growth performance. This is also reflected in the nearly linear trends fitted on the logit scale for the fitness-performance relationships modelling a quadratic term (Supplementary Figure 1).

Supplementary Table 5 | Relationship of tree survival with the six functional traits across the economic and hydraulic sets, and excluding or including the modelling of growth performance measures (height or diameter), assessed in a common-garden field trial of *E. pauciflora*. The results refer to average directional selection gradients estimated for the studied continuous predictor variables.

	Excluding height or diameter as a predictor variable	Including height as a predictor variable	Including diameter as a predictor variable
Leaf thickness	-0.329 ± 0.757<br>(-1.786, 1.151)	0.704 ± 0.735<br>(-0.648, 2.336)	0.349 ± 0.765<br>(-1.051, 1.949)
Leaf area	0.197 ± 0.234<br>(-0.305, 0.618)	0.137 ± 0.220<br>(-0.326, 0.549)	0.150 ± 0.229<br>(-0.320, 0.574)
Leaf density	1.560 ± 0.837<br>(-0.230, 3.093)	0.424 ± 0.802<br>(-1.078, 2.107)	0.914 ± 0.828<br>(-0.544, 2.795)
Stomatal density	-0.395 ± 0.345<br>(-1.094, 0.258)	-0.133 ± 0.413<br>(-0.829, 0.805)	-0.262 ± 0.387<br>(-0.915, 0.620)
Stomatal length	0.559 ± 0.579<br>(-0.495 1.761)	0.250 ± 0.652<br>(-0.958, 1.563)	0.251 ± 0.593<br>(-0.855, 1.457)
Vein density	-0.031 ± 0.339<br>(-0.660, 0.676)	0.015 ± 0.323<br>(-0.579, 0.708)	0.017 ± 0.333<br>(-0.594, 0.723)
Height	-	0.940 ± 0.286<br>*(0.489, 1.458)*	-
Diameter	-	-	0.350 ± 0.114<br>*(0.136, 0.578)*

The growth performance measures and the functional traits were assessed at age 2 years from planting, and tree survival (considered as a fitness component) was assessed at age 7 years. Tree survival was scored as a binary response, which was modelled with a (fixed-effects) generalized linear model using the logit link function to relate the conditional expectation of the response variable to a linear predictor. Besides incorporating populations and blocks as classification predictor variables, the linear predictor also comprised the six functional traits across the economic and hydraulic sets as covariates and, in both cases, it excluded or included a given growth performance variable (height or diameter). Modelling performance as a covariate in the linear predictor allows estimating the (direct) effects of functional traits on tree survival that are not mediated by growth performance (e.g. see Franklin and Morrissey, 2017). Estimated average directional selection gradients, and their standard errors, are presented for the studied continuous predictor variables, and can be interpreted as the percentage change (on average) in expected relative survival from increasing the focal covariate values by 1%, conditional on the observed values of the other explanatory variables involved in the definition of the linear predictor (see Supplementary Methods 2). A non-parametric cluster bootstrapping procedure was pursued to obtain the standard error for a selection gradient estimate, as well as to provide the corresponding bias-corrected and accelerated (BCa) 95% confidence interval (within parentheses) (see Supplementary Methods 3). Statistical support against a null hypothesis being true (i.e. against the absence of an effect) is given by a 95% BCa confidence interval not overlapping with zero (indicated in Italics).

Supplementary Table 6 | Relationship of tree survival with leaf economic and hydraulic traits, and excluding or including the modelling of growth performance measures (height or diameter), assessed in a common-garden field trial of *E. pauciflora*. The results refer to estimated coefficients of regression parameters on the logit (log-odds) scale.

	Excluding height or diameter as a predictor variable	Including height as a predictor variable	Including diameter as a predictor variable
(a) Analyses of leaf economic traits			
Tjur's coefficient of discrimination	0.316	0.489	0.392
Leaf thickness	-0.0215<br>(-0.0655, 0.0200)	0.0375<br>(-0.0151, 0.0931)	0.0158<br>(-0.0339, 0.0647)
Leaf area	0.0062<br>(-0.0127, 0.0257)	0.0052<br>(-0.0158, 0.0267)	0.0055<br>(-0.0140, 0.0258)
Leaf density	0.0688<br>*(0.0063, 0.1373)*	0.0211<br>(-0.0598, 0.1075)	0.0407<br>(-0.0277, 0.1147)
Height	-	0.0506<br>*(0.0291, 0.0778)*	-
Diameter	-	-	0.0183<br>*(0.0078, 0.0303)*
(b) Analyses of leaf hydraulic traits			
Tjur's coefficient of discrimination	0.318	0.466	0.382
Stomatal density	-0.0283<br>(-0.0595, 0.0004)	-0.0073<br>(-0.0442, 0.0281)	-0.0158<br>(-0.0503, 0.0171)
Stomatal length	0.0087<br>(-0.0356, 0.0539)	0.0071<br>(-0.0475, 0.0618)	0.0011<br>(-0.0468, 0.0489)
Vein density	-0.0044<br>(-0.0247, 0.0157)	-0.0045<br>(-0.0276, 0.0184)	-0.0027<br>(-0.0238, 0.0185)
Height	-	0.0418<br>*(0.0233, 0.0645)*	-
Diameter	-	-	0.0152<br>*(0.0057, 0.0259)*
(c) Analyses of all functional traits			
Tjur's coefficient of discrimination	0.350	0.490	0.395
Leaf thickness	-0.0133<br>(-0.0631, 0.0340)	0.0343<br>(-0.0229, 0.0944)	0.0150<br>(-0.0394, 0.0689)
Leaf area	0.0080<br>(-0.0123, 0.0289)	0.0067<br>(-0.0151, 0.0293)	0.0064<br>(-0.0139, 0.0280)
Leaf density	0.0633<br>(-0.0009, 0.1335)	0.0207<br>(-0.0593, 0.1033)	0.0392<br>(-0.0291, 0.1125)
Stomatal density	-0.0160<br>(-0.0511, 0.0174)	-0.0065<br>(-0.0473, 0.0335)	-0.0112<br>(-0.0490, 0.0262)
Stomatal length	0.0227<br>(-0.0258, 0.0739)	0.0122<br>(-0.0475, 0.0728)	0.0108<br>(-0.0413, 0.0647)
Vein density	-0.0013<br>(-0.0227, 0.0202)	0.0007<br>(-0.0232, 0.0257)	0.0007<br>(-0.0212, 0.0231)
Height	-	0.0458<br>*(0.0243, 0.0731)*	-
Diameter	-	-	0.0150<br>*(0.0042, 0.0274)*

The growth performance measures and the functional traits were assessed at age 2 years from planting, and tree survival was assessed at age 7 years. Tree survival was scored as a binary response, which was modelled with a (fixed-effects) generalized linear model using the logit link function to relate the conditional expectation of the response variable to a linear predictor. Besides incorporating populations and blocks as classification predictor variables, in *(a)*, *(b)* and *(c)*, the linear predictor also comprised leaf economic traits, leaf hydraulic traits and all functional traits, respectively, as covariates; in all cases, it excluded or included a given performance variable (height or diameter). The results are provided for survival regressed on relative values (i.e. percentage values following mean-standardization by using the grand means of the traits or performance measures) of the covariate observations. Therefore, the tabulated coefficient of a regression parameter refers to the change in the log-odds of the response (i.e. the log-odds that survival equals one) for a 1% increase in the focal covariate, while holding the other modelled explanatory variables constant. The estimation of the model parameters was based on the bias-reducing penalized maximum likelihood method described by Firth (1993); 95% profile penalized-likelihood confidence intervals are shown within parentheses. Statistical support against a null hypothesis being true (i.e. against the absence of a covariate effect on the log-odds of survival) is given by a 95% confidence interval not overlapping with zero (indicated in Italics). The Tjur's coefficient of discrimination (Tjur, 2009) is presented as a measure of the predictive ability of the model; as a reference value, a logistic model comprising only populations and blocks as fixed-effects parameters in the linear predictor resulted in a Tjur's coefficient of discrimination of 0.269.

Supplementary Table 7 | Estimated within-population raw and partial correlations between specific leaf area and each of three leaf economic traits (leaf thickness, leaf area and leaf density), assessed in a common-garden field trial of *E. pauciflora*.

	Leaf thickness	Leaf area	Leaf density
Raw correlations	-0.772<br>($p < 0.001$)	-0.199<br>($p = 0.018$)	-0.442<br>($p < 0.001$)
Partial correlations	-0.908<br>($p < 0.001$)	0.063<br>($p = 0.472$)	-0.817<br>($p < 0.001$)

All the functional traits were assessed at age 2 years from planting. A tabulated within-population raw correlation was calculated from a bivariate analysis of a given pair of traits, under a (fixed-effects) linear model that was specified with populations and blocks as classification predictors. In addition to these variables, the bivariate model for the calculation of a within-population partial correlation between specific leaf area and a given trait also included the remaining two leaf economic traits as continuous predictors. Thus, a partial correlation quantifies the strength of the relationship between specific leaf area and a leaf economic trait, while controlling the effect of the other two traits. Estimation of the correlation coefficients and associated statistical inference (*p*-values within parentheses) were provided by using the MANOVA statement under the GLM procedure of SAS (SAS, 2017).

Supplementary Table 8 | Relationship of growth performance measured by tree height or diameter with specific leaf area and leaf area, assessed in a common-garden field trial of *E. pauciflora*.

Growth performance measure	Specific leaf area	Leaf area
(a) Single-predictor specification		
Height	0.088 ± 0.236<br>(-0.379, 0.556)<br>p = 0.708	-0.013 ± 0.107<br>(-0.225, 0.198)<br>p = 0.900
Diameter	0.306 ± 0.384<br>(-0.453, 1.065)<br>p = 0.427	-0.039 ± 0.174<br>(-0.384, 0.305)<br>p = 0.822
(b)Multiple-predictor specification		
Height	0.086 ± 0.242<br>(-0.392, 0.564)<br>p = 0.723	-0.006 ± 0.110<br>(-0.222, 0.211)<br>p = 0.958
Diameter	0.301 ± 0.393<br>(-0.477, 1.078)<br>p = 0.446	-0.012 ± 0.178<br>(-0.364, 0.340)<br>p = 0.946

The growth performance measures and the functional traits were assessed at age 2 years from planting. A given growth performance measure was modelled as the dependent variable in a (fixed-effects) linear model including populations and blocks as classification predictors, and functional traits as continuous predictors according to the following specifications: in *(a)*, each functional trait was individually modelled as a predictor variable; and in *(b)*, the two functional traits were simultaneously modelled as predictor variables. The table presents the regression coefficients estimated for the traits under a given model specification, together with the associated standard errors and 95% confidence intervals (within parentheses). Also provided are the p-values of statistical t-tests undertaken to evaluate whether a regression coefficient estimate differed significantly from zero. A regression coefficient estimate refers to the percentage change in relative performance (i.e. height or diameter change in % of the mean) expected from increasing the functional trait by 1%, while holding the other explanatory variables modelled constant. Note that, for leaf area under the "Single-predictor specification", the results are the same as those shown in Table 2 of the article.

4. Supplementary references

Allison, P.D. (2009). *Fixed Effects Regression Models*. London: Sage.

Arnold, S.J. (1983). Morphology, performance and fitness. *American Zoologist* 23, 347-361.

Arnold, S.J. (2003). Performance surfaces and adaptive landscapes. *Integrative and Comparative Biology*, 43, 367-375.

Bell, A., and Jones, K. (2015). Explaining fixed effects: random effects modelling of time-series cross-sectional and panel data. *Political Science Research and Methods* 3, 133-153.

Bell, A., Jones, K., and Fairbrother, M. (2018). Understanding and misunderstanding group mean centering: a commentary on Kelley et al.'s dangerous practice. *Quality and Quantity* 52, 2031-2036.

Bell, A., Fairbrother, M., and Jones, K. (2019). Fixed and random effects models: making an informed choice. *Quality and Quantity* 53, 1051-1074.

Breen, R., Karlson, K.B., and Holm, A. (2018). Interpreting and understanding logits, probits, and other nonlinear probability models. *Annual Review of Sociology* 44, 39-54.

Cameron, A.C., and Trivedi, P.K. (2010). *Microeconometrics Using Stata (Revised Edition)*. College Station: Stata Press.

Clark, T.S., and Linzer, D.A. (2015). Should I use fixed or random effects? *Political Science Research and Methods* 3, 399-408.

Davison, A. C., and Hinkley, R. (1997). *Bootstrap Methods and Their Applications*. Cambridge: Cambridge University Press.

Efron, B., and Tibshirani, R.J. (1993). *An Introduction to the Bootstrap*. New York: Chapman & Hall.

Fairbrother, M. (2014). Two multilevel modeling techniques for analyzing comparative longitudinal survey datasets. *Political Science Research and Methods* 2, 119-140.

Firth, D. (1993). Bias reduction of maximum likelihood estimates. *Biometrika* 80**,** 27-38.

Franklin, O.D., and Morrissey, M.B. (2017). Inference of selection gradients using performance measures as fitness proxies. *Methods in Ecology and Evolution* 8, 663-677.

Gleason J.R. (1988). Algorithms for balanced bootstrap simulations. *The American Statistician* 42, 263-266.

Goetgeluk, S., and Vansteelandt, S. (2008). Conditional generalized estimating equations for the analysis of clustered and longitudinal data. *Biometrics* 64, 772-780.

Janzen, F.J., and Stern, H.S. (1998). Logistic regression for empirical studies of multivariate selection. *Evolution* 52, 1564-1571.

Kenward, M.G., and Roger, J.H. (1997). Small sample inference for fixed effects from restricted maximum likelihood. *Biometrics* 53, 983-997.

Kenward, M.G., and Roger, J.H. (2009). An improved approximation to the precision of fixed effects from restricted maximum likelihood. *Computational Statistics and Data Analysis* 53, 2583-2595.

Lande, R. (1979). Quantitative genetic analysis of multivariate evolution, applied to brain: body size allometry. *Evolution* 33, 402-416.

Lande, R., and Arnold, S.J. (1983). The measurement of selection on correlated characters. *Evolution* 37, 1210-1226.

Morrissey, M.B., and Sakrejda, K. (2013). Unification of regression-based methods for the analysis of natural selection. *Evolution* 67, 2094-2100.

Patterson, H.D., and Thompson, R. (1971). Recovery of inter-block information when block sizes are unequal. *Biometrika* 58, 545-554.

Raudenbush, S.W., and Bryk, A.S. (2002). *Hierarchical linear models: Applications and data analysis methods.* Thousand Oaks, CA: Sage Publications.

SAS Institute Inc. (2017). SAS/STAT® 14.3 User's Guide. Cary, NC: SAS Institute Inc.

Shapiro, S.S., and Wilk, M.B. (1965). An analysis of variance test for normality (complete samples). *Biometrika* 52, 591-611.

Tjur, T. (2009). Coefficients of determination in logistic regression models - a new proposal: the coefficient of discrimination. *The American Statistician* 63**,** 366-372.

Van der Leeden, R., Meijer, E., and Busing, F. (2008). Resampling multilevel models. *In*: J. de Leeuw and E. Meijer (Eds.), *Handbook of Multilevel Analysis*, Chapter 11, pp. 401-433. New York: Springer.

Walsh, B., and Lynch, M. (2018). *Evolution and Selection of Quantitative Traits*. Oxford: Oxford University Press.

Williams, R. (2021). Marginal effects for continuous variables. University of Notre Dame. Working paper, *https://www3.nd.edu/~rwilliam/stats3/Margins02.pdf* , accessed 27 January 2022.
